# Studies of pesticide residues in tomatoes and cucumbers from Kazakhstan and the associated health risks

**DOI:** 10.1007/s10661-015-4818-6

**Published:** 2015-09-04

**Authors:** Bozena Lozowicka, Elmira Abzeitova, Abai Sagitov, Piotr Kaczynski, Kazbek Toleubayev, Alina Li

**Affiliations:** Laboratory of Pesticide Residues, Plant Protection Institute - National Research Institute, Chełmonskiego 22 Str, 15195 Bialystok, Poland; Kazakh National Agrarian University, Abai 8 Str, Almaty, Kazakhstan 050010; Kazakh Research Institute for Plant Protection and Quarantine, Rakhat, Karasay District, Almaty, Kazakhstan 040924; Kostanay State University, A. Baitursynov, Mayakovsky 99/1 Str, Kostanay, Kazakhstan 110000

**Keywords:** Tomatoes and cucumbers, Pesticide residue, Greenhouse, Risk assessment, Kazakhstan

## Abstract

The aim of the present study was to assess the level of pesticide residues in vegetables in the Almaty Region of Kazakhstan and to determine the potential health risks associated with the exposures to these pesticides. A total of 82 samples of cucumbers and tomatoes from top agro-based market and greenhouses were analysed using a gas chromatography–micro electron capture detector/nitrogen–phosphorous detector (GC-μECD/NPD), a multiresidue method to analyse 184 different pesticide types. The results indicated that more than half of samples (59 %) contained 29 pesticides, in which 10 are not registered in Kazakhstan, ranging from 0.01 to 0.88 mg kg^−1^, and 28 % contained pesticide residues above maximum residue levels (MRLs). The estimated daily intakes (EDIs) ranged from 0.01 % of the acceptable daily intake (ADI) for pyrimethanil to 12.05 % of the ADI for lambda-cyhalothrin. The most critical commodity is triazophos and flusilazole in tomatoes, contributing 70.8 and 42.5 % to the acute hazard index (aHI). The results provided important information on the current pesticide contamination status of two commonly consumed vegetables and pointed an urgent need to control the use of plant protection products applied, especially potentially persistent pesticides, such as endosulfan and dicofol. These results also show that the detected pesticides may be considered a public health problem.

**Electronic supplementary material:**

The online version of this article (doi:10.1007/s10661-015-4818-6) contains supplementary material, which is available to authorized users.

## Introduction

Since 2008, the government of Kazakhstan has revived and invested into the greenhouse industry to satisfy the increasing demand for vegetables. In the end of 2013, approximately 800 ha of sheltered ground was provided for vegetable production, mainly for cucumbers and tomatoes (an internal estimate from various sources because of conflicting statistics). In Kazakhstan, tomatoes (*Lycopersicon esculentum* Mill.) and cucumbers (*Cucumis sativus*) are some of the most important vegetable components of the diet and are consumed raw, cooked or processed. Nevertheless, tomato and cucumber plants are susceptible to several pests and diseases that have been controlled with pesticides to avoid significant yield losses.

Based on our monitoring of 44 greenhouse farms in Kazakhstan during 2012–2014, we conclude that producers are facing severe pest and fungi problems. To combat pests, they frequently apply various insectoacaricides, sometimes of unknown nature and origin and at increased dosages. We also witnessed pesticide treatments being performed just before harvesting and marketing.

This application of pesticides happens despite the fact that Kazakhstan has an official list of pesticides permitted for use on various crops against different agrophages in open fields and sheltered ground with defined dosages, frequencies of application and expected time before harvest. For use in greenhouses, insectoacaricides with the following active ingredients are officially allowed: abamectin, bensultap, cypermethrin imidacloprid, lambda-cyhalothrin, pirimiphos-methyl, рropargite and thiamethoxam. The active substances permitted as fungicides in greenhouses are the following: copper sulfate and boscalid, chlorothalonil, folpet, iprodione, metiram, pyraclostrobin, triadimefon and triadimenol (Ministry of Agriculture of the Republic of Kazakhstan 2012). However, select information provided in this official document produces many questions (Nazhmetidinova 2001). For instance, pirimiphos-methyl is registered to be used in open fields and greenhouses on cucumbers and tomatoes and applied against whiteflies, mites, aphids and thrips. The maximum application frequency is identical for both situations which is two times. However, its dosage for open field applications is 0.3–1.5 L ha^−1^, but for sheltered ground applications, its dosage is 3.0–5.0 L ha^−1^. The expected time before harvest after the last application is 20 days in an open field and 3 days in greenhouses.

Apart from the vegetables of local greenhouse producers, Kazakhstan imports tomatoes and cucumbers from neighbouring regions of China and Uzbekistan. No monitoring and detection of pesticide residues in imported vegetables are performed at the point of entry or in marketing places, and no certificate of origin is provided by local retailers; another problem is the lack of pesticide residue monitoring in soils. Additionally, farmers are not obliged to report pesticides used; that is why the history of plant protection product application is very often difficult to trace.

A good diet rich in vegetables has been shown to be an important factor in reducing the risk of diseases. The consumption of tomatoes and cucumbers may be important in prostate and pancreatic cancer prevention. These vegetables contain all four major carotenoids (alpha- and beta-carotene, lutein and lycopene) and all three high-powered antioxidants (beta-carotene which has vitamin A activity in the body, vitamin E and vitamin C), may have individual benefits and display synergy as a group (that is, they interact to provide health benefits).

Pesticide residues on vegetables constitute a possible risk to consumers and have been a human health concern. When a chemical is used as recommended on the label of the product, any residues that occur should not exceed the maximum residue levels (MRLs). Residues detected in excess of the MRL rarely constitute a toxicological concern. A good knowledge of the pesticide concentration is necessary to properly assess human exposure. Health risk assessment of pesticide residues in contaminated vegetables is performed in developed countries (Akoto et al. 2013; EFSA 2013); however, these residues are minimally explored in developing countries (Vieira et al. 2014).

There is a lack of scientific works in the literature that describe the level of contamination of main vegetables produced and consumed in Kazakhstan with multi-class pesticide. Only a small number of works are related to determination of certain active substances in agricultural products (Lozowicka et al. 2013). The agricultural practices in this country are almost absent because of the lack of a correct pest management system and pesticide laws, and the risk to human health, exposure to pesticide residues and types of health threats must be evaluated. The aim of this study was to measure the level of pesticide residues present in samples of tomatoes and cucumbers produced in Kazakhstan during 3 years (2012–2014) and to evaluate the human health implications of pesticide residues in vegetables. In studies, we investigated over 180 active substances: insecticides, fungicides, herbicides and acaricides. Analyses were carried out in a Polish scientific laboratory that possesses an implemented ISO/IEC 17025:2005 system. For this purpose, a multi-method based on matrix solid phase dispersion and a gas chromatography technique with a dual-detection system (electron capture detector/nitrogen–phosphorous detector) were applied. Pesticide residue levels were evaluated in relation to acceptable daily intakes (ADIs), acute reference doses (ARfDs) derived from toxicological studies and MRLs (EC 2005; CU 2010).

## Material and methods

### Samples and reagents

**Fig. 1:**
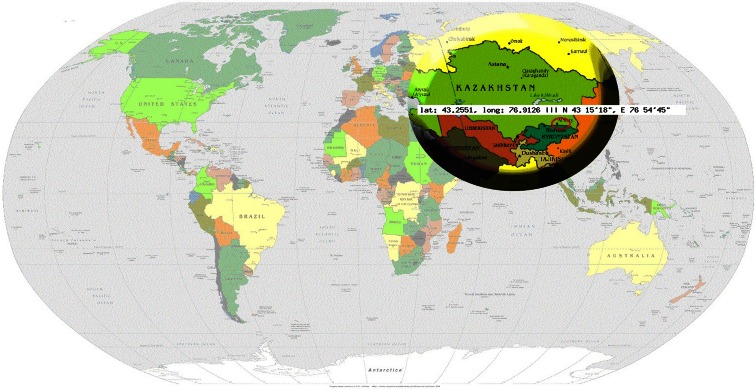
Collected samples from Almaty, Kazakhstan

In this study, 82 samples were collected in 2012–2014 (April, November and December) (44 tomato and 38 cucumber samples) from Almaty (former capital of Kazakhstan situated at 43.25° north latitude, 76.95° east longitude; Kazakhstan, Asia) (Fig. 1). This place has 2 greenhouses, 5 supermarkets and 11 open markets. We state that sampled vegetables are of greenhouse origin since by the end of April, November and December, climatic conditions in Kazakhstan are unsuitable for open field cultivation. Samples of pesticide-free organic cucumbers and tomatoes (additionally, previously were checked for present of pesticide residues) were used as blank to spike for the validation process.

All reagents used were of analytical grade. Acetone, *n*-hexane and diethyl ether for pesticide residue analysis were provided by J.T.Baker (Deventer, Holland). Sodium sulfate anhydrous (Fluka, Seelze, Hannover, Germany) and silica gel (Merck, Darmstadt, Germany) were activated 8 h at 600 °C. Silica gel was deactivated before analysis and prepared by adding 5 mL of water to 95 g of activated silica gel.

### Standards

Pesticides (64 fungicides, 26 herbicides and 94 insecticides) were obtained from the Dr. Ehrenstorfer Laboratory (Germany). Pesticide standard stock solutions (>95 % purity for all standards ) of various concentrations were prepared in acetone and stored at 4 °C. Standard working solutions were prepared by dissolving appropriate amounts of stock solution with a mixture of hexane/acetone (9:1, *v*/*v*).

### Sample preparation

A representative portion of the cucumbers or tomatoes was blended. Additionally, 2.5 mL of 1 % H_2_SO_4_ was added to 10 g of cucumbers. 2.0 g of the sample was put in a mortar with 4.0 g activated silica gel (cucumbers) or 5 % deactivated silica gel (tomatoes) and was manually mixed using a pestle to produce a homogeneous mixture (4 min). The mixed material was transferred to the glass column (1.5 cm in i.d. × 40 cm in length) containing anhydrous sodium sulfate (5.0 g) and activated silica gel (2.5 g). Anhydrous sodium sulfate (5.0 g) on the top was added. The analytes were eluted using 30 mL of a mixture of 15 mL hexane/acetone (8:2, *v*/*v*) and 15 mL hexane/ethyl ether/acetone (1:2:2, *v*/*v*/*v*). The extract was evaporated at a temperature of 40 °C and then diluted in 2 mL of hexane/acetone (9:1, *v*/*v*). One millilitre of the final solution was put into a gas chromatography (GC) vessel and placed to the rack of the autosampler.

### Instrumentation and chromatographic conditions

GC analysis was performed with an Agilent (Waldbronn, Germany) model 7890A gas chromatograph equipped with electron capture detector (ECD) and nitrogen–phosphorus detector (NPD) with mid-polarity DB-1701 column (14 % (cyanopropylphenyl) methylsiloxane phase, 15 m × 0.53 mm × 1 μm film), and ChemStation chromatography manager data acquisition and processing system (Hewlett-Packard, version A.10.2). For confirmation of residues, a DB-35 column (35 % (phenyl) methylpolysiloxane phase; 30 m × 0.32 mm and film thickness 0.50 μm) was used. The operating conditions for DB-1701 column (1) and DB-35 column (2) were as follows: for detectors, injector temperature of 210 °C (1 and 2); carrier gas, helium at a flow rate of 4.0 mL min^−1^ (1) and 1.9 mL min^−1^ (2); detector temperature of 300 °C (ECD and NPD) (1 and 2); make-up gas: nitrogen at a flow rate of 55 mL min^−1^ (1) and 60 mL min^−1^ (2) (ECD) and 25 mL min^−1^ (1) and 8 mL min^−1^ (2) (NPD); hydrogen at a flow rate of 3.5 mL min^−1^ (1) and 3.0 mL min^−1^ (2); and air at a flow rate of 70 mL min^−1^ (1) and 60 mL min^−1^ (2); and for oven (1), initial temperature of 120 °C at 16 °C min^−1^ up to 190 °C, 190 °C at 8 °C min^−1^ up to 230 °C, 230 °C at 18 °C min^−1^ up to 260 °C and held for 12.57 min at the final temperature and, for oven (2), initial temperature of 120 °C at 13 °C min^−1^ up to 190 °C, 190 °C at 8 °C min^−1^ up to 240 °C, 240 °C at 16 °C min^−1^ up to 295 °C and held for 20.0 min at the final temperature. The volume of final sample extract injected at 210 °C in splitless mode (purge off time 2 min) was 2 μL injected, and the peak height was compared to that of the calibration standards (in matrices) to determine the residue quantitatively.

### Method of validation

In this study, both organic cucumbers and tomatoes were selected as a commodity for the validation of the method (EC 2009, 2013) in determination of pesticide residue (Supplementary Material Table S1).

#### Preparation of calibration standards

Calibration curves were obtained from matrix-matched calibration solutions. The lowest concentration level in the calibration curve was established as a limit of detection. Calibration standards were prepared by addition of appropriate spiking solutions to a blank matrix of the cucumber and tomatoes to produce a final concentration of first range 0.001–0.05 mg kg^−1^, second range 0.05–0.5 mg kg^−1^ and third range 0.5–2.5 mg kg^−1^.

#### Recovery studies

Recovery data was obtained at the three speaking levels of pesticides in the matrix each day using blank cucumber and tomato samples in accordance with European Commission (EC) guidelines (EC 2009, 2013). Blank samples (2.0 g) after homogenization were spiked by addition of appropriate volumes of pesticide standard mixture in hexane/acetone (9:1, *v*/*v*) solution and were left for 1 h (equilibration times) and then prepared according to the procedures described in the “Sample preparation” section. Method accuracy and precision were evaluated by performing recovery studies. The precision was expressed as the relative standard deviation (RSD). Accuracy can be measured by analyzing the samples with known concentration and comparing the measured values with the true values.

#### Limit of quantitation and limit of detection

The limit of quantification (LOQ) was defined as the lowest concentration of the analyte that could be quantified with acceptable precision and accuracy. The limit of detection (LOD) was defined as the lowest concentration of the analyte in a sample which could be detected but not necessarily quantified. The LOQ and LOD were evaluated as the signal-to-noise ratio (S/N) of 10:1 and 3:1 for the pesticide, respectively.

### Quality check

To be sure about the quality of results, the laboratory has accreditation PN/EN ISO IEC 17025 (from 2007) (ISO 2005) and regularly takes a part and satisfactory performance in external proficiency assessment schemes in proficiency testing schemes organised and run by the Food Analysis Performance Assessment Scheme (FAPAS; Central Science Laboratory in York) and by the European Commission (University of Almeria). Participation in EC tests is mandatory for all official laboratories undertaking the analysis of these commodities for the official controls on pesticide residues, use of validated methods and the employment of suitably qualified persons to carry out analysis (Supplementary Material Table S2).

## Health risk estimation

The health risk estimation was calculated through a comparison of the found residues to the established acceptable daily intake (ADI) and acute reference dose (ARfD) values (Renwick 2002). The residue concentration in a product was determined as the arithmetic mean of all the results obtained. The results under the LOD of the analytical methods used for the intake calculations were considered as LOD values. The values of ADI and ARfD were elaborated by the Joint FAO/FAO Meeting on Pesticides Residues (WHO/FAO 2002), European Food Safety Authority (EFSA 2009) of the European Union and the Federal Institute for Risk Assessment (BfR), Germany (BfR 2006). The short-term (acute) and long-term (chronic) dietary consumer exposure to pesticide residues was estimated using an EFSA calculation model, the Pesticide Residue Intake Model ‘PRIMo’ revision 2 (EFSA 2008; Heusinkveld et al. 2013), that was based on the national food consumption and unit weights. The model implements internationally agreed risk assessment methodologies to assess the acute and chronic exposure of consumers, accepting consumption at the 97.5th percentile level (GEMS/Food 2012). Currently, four different situations are distinguished in the International Estimate of Short-Term Intake (IESTI) calculation; each situation maintains a specific mathematical method depending on the unit weights of the commodity (case 1, cases 2a and 2b, case 3). For tomatoes and cucumbers, the methodology used is described in cases 2a and 2b. For cases 2a and 2b, the portion (meal size), e.g. a piece of vegetable, may contain a higher residue than the composite samples from residue trials (unit weight >25 g). A variability factor depending on the properties of a product is therefore introduced (a standard factor is based on the available residue data in separate pieces of fruits or vegetables). Specifics for case 2a (tomatoes), IESTI = [{*U* × HR – P × *v*} + {(LP − *U*) × HR – P}]/b . w., and specifics for case 2b (cucumbers), IESTI = LP × (HR – P) × *v*/b . w., where *U* is the unit weight of individual items of the commodity in kilograms, HR–P is the highest residue level in milligrams per kilogram, *v* is the variability factor applied to the composite residue to approximate the residue level in a high-residue single unit (depending on the commodity, cucumbers: *v* = 5 and tomatoes: *v* = 7), LP (large portion) is the 97.5th percentile of portion sizes of people consuming the commodity in kilograms of food per day, and b.w. is the mean body weight for the target population subgroup in kilograms. The IESTI is compared to the ARfD for the pesticide, and the acute hazard index was calculated as follows: aHI = IESTI/ARfD. When the IESTI is less than the ARfD, the risk is considered acceptable, and when the IESTI exceeds the ARfD, the risk is considered unacceptable.

The International Estimated Daily Intake (IEDI) of pesticide residues was calculated as follows: $\mathrm{IEDI}={\displaystyle \sum \left({F}_i\times {RL}_i\right)/\mathrm{mean}\ \mathrm{b}.\mathrm{w}.}$ , where *F*_*i*_ is the food consumption data, and RL_*i*_ is the residue level of the commodity. The long-term risk assessment of the intakes compared to the pesticide toxicological data was performed for clusters B and D, adults and children by calculating the hazard quotient (HQ). This process divided the IEDI with the relevant acceptable daily intake that is considered to be a safe exposure level over the lifetime: HQ = IEDI/ADI × 100% , where ADI is the acceptable daily intake. The HQ was calculated both for pesticides and commodities. The HQs are summed to produce a chronic hazard index (cHI): $\mathrm{cHI}={\displaystyle \sum HQ}$.

## Results and discussion

### Study of the validation method

The parameters used to validate the method were the matrix effect, linearity, precision and accuracy, sensibility (limits of detection and quantification) and repeatability. All analyses were performed using the pesticide-free organic tomatoes and cucumbers. In total, 184 pesticides were extracted using matrix solid phase dispersion (MSPD) and analysed by gas chromatography (GC) with a dual-detection system: electron capture detector (ECD) and nitrogen–phosphorus (NPD). The linearity was evaluated on a five-point linear plot with three replicates by calculating the linear regression and squared correlation coefficient (*R*^2^). All pesticides displayed a linearity in the concentration range of 0.001–2.5 mg kg^−1^ with correlation coefficients higher than 0.99283 (metazachlor) up to 1. The matrix effect on the detector response for the studied pesticides and matrices was evaluated in the present work. To determine whether a different response was noted between the matrix-matched standards and the standards in the solvent, matrix-matched standards were used. In this study, recovery experiments for the 184 pesticides at three spiking levels (0.001 to 0.05 , 0.05–0.5 and 0.1–2.5 mg kg^−1^) for a period of 5 days were performed. The mean recoveries for tomatoes and cucumbers spiked at three fortification levels ranged from 71.07 to 119.90 %, with the exception of cyazofamid, fenbuconazole, buprofezin, cypermethrin, lambda-cyhalothrin, omethoate and phosalone (40–70 %) and beta-endosulfan, heptachlor, methidathion, *p*,*p*′-dichlorodiphenyltrichloroethane (DDT) and tetraconazole (120–140 %) with RSDs of 0.15–12.8 %. Each pesticide was fortified at its LOQ level, at the maximum residue level (MRL) or at 10 times the LOQ level and at a third intermediate level. However, a range of 60–140 % may be used in routine multiresidue analyses (EC 2009, 2013). Relative standard deviations ranged from 0.10 to 12.8 %, displaying a good repeatability. The accuracy and precision of the method were tested via the recovery experiments with fortified samples. The method precision was expressed as the repeatability (10 replicates) of the recovery at the studied spiked levels, and the RSDs for all compounds have been defined (>20 %). The validation of the parameters (Table S1) and participating in the proficiency testing (Table S2) are presented in the Supplementary Material. These results indicate that the recoveries and accuracy for the pesticides were good and competence of the laboratory was confirmed. Consequently, the pesticides were satisfactorily determined using these methods. The LOD values of individual pesticides were calculated based on the noise level in the chromatograms at S/N of 3:1. The LOQs of the proposed method were calculated by considering a value 10 times that of the background noise. For most compounds, the values are lower than their respective MRLs. The LOQs ranged from 0.001 to 0.004 mg kg^−1^. For all pesticides analysed, the LODs are lower than the respective MRLs established by the European Union and Custom Union regulation for tomatoes and cucumbers (EC 2005; CU 2010).

### Pesticide residues analytical results

**Fig. 2:**
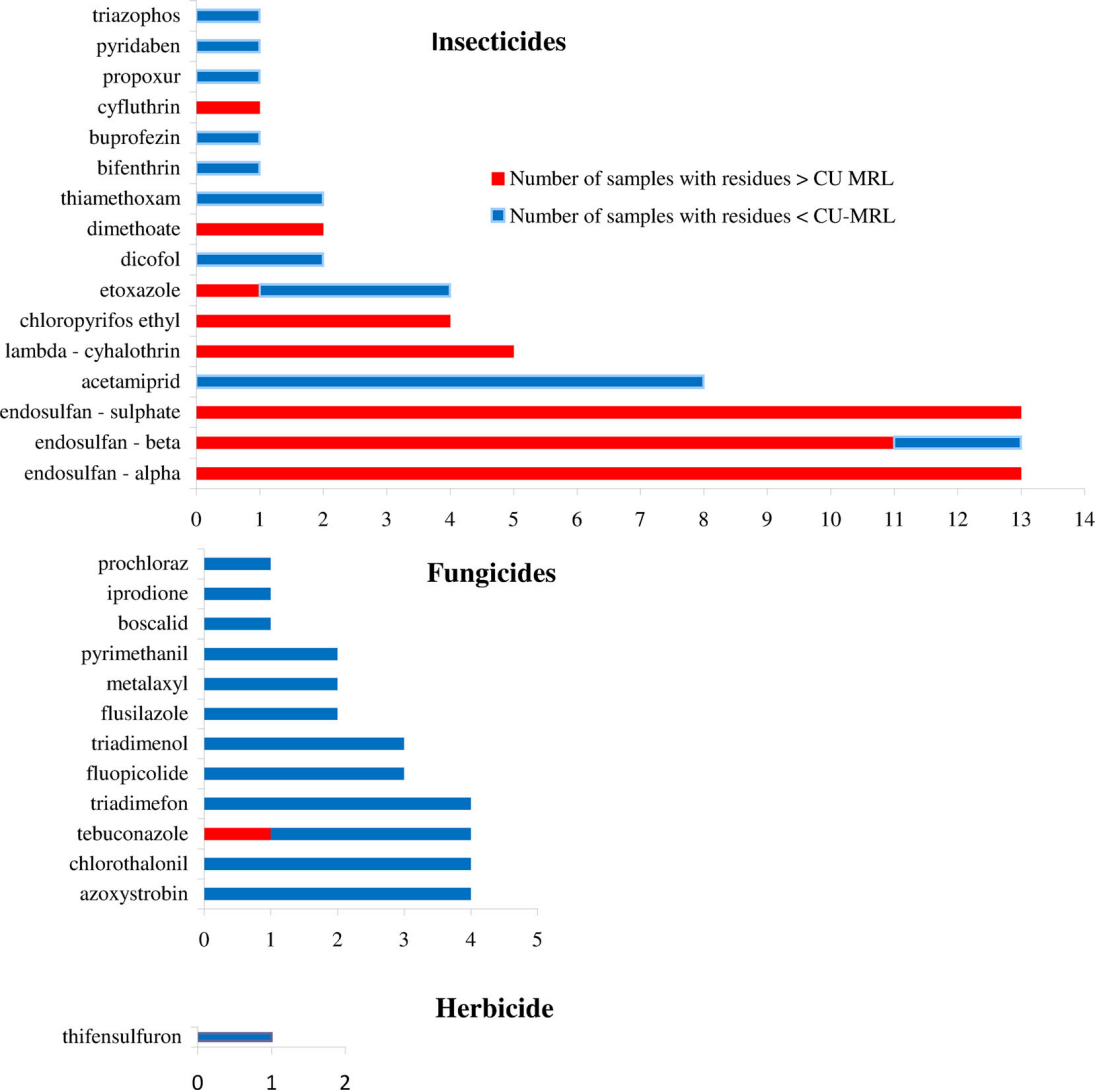
The frequency occurrence of active substances in tomato and cucumber samples

The concentrations of pesticide residues found in vegetables sampled from the local markets and greenhouses of Almaty metropolis in Kazakhstan are summarised in Table 1. The frequency of detected active substances in tomatoes and cucumbers is presented in Fig. 2. Pesticide residues were not observed in 34 (41.5 %) out of the 82 samples analysed. The concentration of all detected pesticide residues found in 48 samples (58.5 %) was compared with the maximum residue levels set by the European Commission (EC 2005) EU-MRLs and Custom Union (Russia, Belorussia and Kazakhstan) (CU 2010) (Tables 1 and 2). According to the unified requirements of the Custom Union (Russia, Belorussia and Kazakhstan), 498 MRLs have been defined for residues of active substances and its metabolites in food products. When no value was defined for residues of active substances, the MRL Codex Alimentarius was used.

**Table 1:** The results of pesticide residues detected in cucumbers and tomato samples from Almaty (2012–2014)

Sample	Pesticide residue	Mode of action	Concentration (mg kg^−1^)	EU-MRL (mg kg^−1^)	CU-MRL (mg kg^−1^)
Cucumbers					
1	Acetamiprid	I	0.250	0.20	0.30
2	Dimethoate	I	0.130	0.02	0.02
3	Acetamiprid	I	0.010	0.20	0.30
	Chlorothalonil	F	0.010	2.00	0.10
	Fluopicolide	F	0.010	1.00	0.01
4	Chlorpyrifos	I	0.070	0.05	0.01
5	Alpha-endosulfan	I	0.004	–	0.002
	Beta-endosulfan	I	0.001	–	0.002
	Endosulfan sulfate	I	0.003	–	0.002
	Σ Endosulfan	I	0.008	0.05	0.002
6	Triadimefon	F	0.020	–	0.50
	Triadimenol	F	0.020	–	0.10
	Σ Triadimefon and triadimenol	F	0.040	0.20	–
	Tebuconazole	F	0.020	0.50	0.20
7	Fluopicolide	F	0.020	1.00	0.05
8	Acetamiprid	I	0.100	0.20	0.30
9	Alpha-endosulfan	I	0.040	–	0.002
	Beta-endosulfan	I	0.020	–	0.002
	Endosulfan sulfate	I	0.020	–	0.002
	Σ Endosulfan	I	0.080	0.05	0.002
	Tebuconazole	F	0.250	0.50	0.01
10	Acetamiprid	I	0.150	0.20	0.30
	Fluopicolide	F	0.030	1.00	0.01
11	Alpha-endosulfan	I	0.005	–	0.002
	Beta-endosulfan	I	0.002	–	0.002
	Endosulfan sulfate	I	0.004	–	0.002
	Σ Endosulfan	I	0.011	0.05	0.002
12	Chlorothalonil	F	0.050	2.00	0.10
	Dimethoate	I	0.050	0.02	0.02
13	Propoxur	I	0.030	0.05	0.01 (CA)
14	Chlorpyrifos ethyl	I	0.030	0.05	0.005
	Lambda-cyhalothrin	I	0.020	0.10	0.01
	Thiamethoxam	I	0.010	0.50	0.20
15	Chlorpyrifos ethyl	I	0.050	0.05	0.005
16	Acetamiprid	I	0.010	0.20	0.30
17	Etoxazole	I	0.030	0.02	0.02 (CA)
18	Thifensulfuron	H	0.010	0.01	0.01 (CA)
	Etoxazole		0.040	0.02	0.02 (CA)
19	Azoxystrobin	F	0.010	1.00	0.20
Tomatoes					
20	Dicofol	A	0.080	0.02	0.10
21	Alpha-endosulfan	I	0.030	–	0.002
	Beta-endosulfan	I	0.020	–	0.002
	Endosulfan sulfate	I	0.010	–	0.002
	Σ Endosulfan	I	0.060	0.05	0.002
22	Metalaxyl	F	0.050	0.50	0.50
	Chlorothalonil	F	0.050	2.00	0.15
23	Alpha-endosulfan	I	0.030	–	0.002
	Beta-endosulfan	I	0.020	–	0.002
	Endosulfan sulfate	I	0.010	–	0.002
	Σ Endosulfan	I	0.060	0.05	0.002
24	Alpha-endosulfan	I	0.040	–	0.002
	Beta-endosulfan	I	0.030	–	0.002
	Endosulfan sulfate	I	0.010	–	0.002
	Σ Endosulfan	I	0.080	0.05	0.002
25	Cyfluthrin	I	0.030	0.02	0.002
26	Chlorothalonil	F	0.060	2.00	0.15
27	Acetamiprid	I	0.020	0.20	0.30
	Alpha-endosulfan	I	0.120	–	0.002
	Beta-endosulfan	I	0.620	–	0.002
	Endosulfan sulfate	I	0.060	–	0.002
	Σ Endosulfan	I	0.880	0.05	0.002
	Lambda-cyhalothrin	I	0.020	0.10	0.01
	Tebuconazole	F	0.020	0.50	0.01
28	Acetamiprid	I	0.040	0.20	0.30
29	Pyrimethanil	F	0.100	1.00	0.70 (CA)
30	Alpha-endosulfan	I	0.040	–	0.002
	Beta-endosulfan	I	0.020	–	0.002
	Endosulfan sulfate	I	0.010	–	0.002
	Σ Endosulfan	I	0.070	0.05	0.002
31	Alpha-endosulfan	I	0.100	–	0.002
	Beta-endosulfan	I	0.100	–	0.002
	Endosulfan sulfate	I	0.080	–	0.002
	Σ Endosulfan	I	0.280	0.05	0.002
32	Lambda-cyhalothrin	I	0.250	0.10	0.01
33	Alpha-cypermethrin	I	0.090	0.50	0.20
34	Triadimefon	F	0.010	–	0.50
	Triadimenol	F	0.010	–	0.10
	Σ Triadimefon and triadimenol	F	0.020	1.00	–
35	Acetamiprid	I	0.180	0.20	0.30
36	Dicofol	A	0.060	0.02	0.10
37	Alpha-cypermethrin	I	0.100	0.50	0.20
38	Alpha-endosulfan	I	0.090	–	0.002
	Beta-endosulfan	I	0.040	–	0.002
	Endosulfan sulfate	I	0.020	–	0.002
	Σ Endosulfan	I	0.150	0.05	0.002
39	Lambda-cyhalothrin	I	0.020	0.10	0.01
40	Chlorpyrifos ethyl	I	0.010	0.50	0.005
	Bifenthrin	IA	0.020	0.30	0.40
41	Triazophos	IA	0.010	0.01	–
	Alpha-endosulfan	I	0.030	–	0.002
	Beta-endosulfan	I	0.020	–	0.002
	Endosulfan sulfate	I	0.008	–	0.002
	Σ Endosulfan	I	0.060	0.05	0.002
	Pyridaben	IA	0.050	0.30	0.20
	Thiamethoxam	I	0.020	0.20	0.20
	Boscalid	F	0.015	3.00	3 (CA)
42	Tebuconazole	F	0.020	0.50	0.01(CA)
43	Iprodione	F	0.030	5.00	5.00
	Prochloraz	F	0.020	0.05	0.01(CA)
44	Azoxystrobin	F	0.020	3.00	0.20
45	Azoxystrobin	F	0.020	3.00	0.20
	Alpha-cypermethrin	I	0.040	0.50	0.005
	Lambda-cyhalothrin	I	0.050	0.10	0.01
	Flusilazole	F	0.300	0.02	0.01 (CA)
	Etoxazole		0.050	0.10	–
46	Azoxystrobin	F	0.020	3.00	0.20
	Metalaxyl	F	0.150	0.50	0.50
	Buprofezin	I	0.170	1.00	0.20
	Alpha-endosulfan	I	0.030	–	0.002
	Beta-endosulfan	I	0.020	–	0.002
	Endosulfan sulfate	I	0.008	–	0.002
	Σ Endosulfan	I	0.060	0.05	0.002
	Flusilazole	F	0.100	0.02	0.01 (CA)
	Triadimefon	F	0.020	–	0.50
	Etoxazole	I	0.020	0.10	–
47	Alpha-endosulfan	I	0.040	–	0.002
	Beta-endosulfan	I	0.020	–	0.002
	Endosulfan sulfate	I	0.010	–	0.002
	Σ Endosulfan	I	0.070	0.05	0.002
	Triadimefon	F	0.040	–	0.50
	Triadimenol	F	0.020	–	0.10
	Σ Triadimefon and triadimenol	F	0.060	1.00	–
48	Acetamiprid	I	0.080	0.20	0.30
	Pyrimethanil	F	0.070	1.00	0.70 (CA)

– no MRLs are available currently

*EU* European Union, *CU* Custom Union, *CA* Codex Alimentarius, *Pest Type*: *I* insecticide, *F* fungicide, *A* acaricide, *H* herbicide

**Table 2:** Compliance of concentration ranges of detected pesticides in cucumber and tomato samples vs. MRLs (European Union and Custom Union)

Group	Active substance	Concentration		EU-MRL (mg kg^−1^)	No. of samples ≤ EU-MRL	No. of samples ≥ EU-MRL	CU-MRL (mg kg^−1^)	No. of samples ≤ CU-MRL	No. of samples ≥ CU-MRL
		Min (mg kg^−1^)	Max (mg kg^−1^)						
Cucumbers									
Insecticides									
Organochlorine	Alpha-endosulfan	0.004	0.04	0.05	3	0	0.002	0	3
	Beta-endosulfan	0.001	0.02	0.05	3	0	0.002	2	1
	Endosulfan sulfate	0.003	0.02	0.05	3	0	0.002	0	3
Neonicotinoid	Acetamiprid	0.01	0.15	0.30	4	0	0.30	4	0
	Thiamethoxam	0.01	0.01	0.05	1	0	0.20	1	0
Organophosphorus	Dimethoate	0.05	0.13	0.02	0	2	0.02	0	2
	Chlorpyrifos ethyl	0.03	0.07	0.05	1	2	0.005	0	3
Pyrethroid	Lambda-cyhalothrin	0.02	0.02	0.10	1	0	0.01	0	1
*N*-Methyl carbamate	Propoxur	0.03	0.03	0.05	2	0	–	0	1
Unclassified	Etoxazole	0.03	0.04	0.02	0	2	–	1	0
Fungicides									
Azole	Tebuconazole	0.20	0.25	0.50	1	0	0.20	0	2
	Triadimefon	0.02	0.02	0.20	1	0	0.50	1	0
	Triadimenol	0.02	0.02	0.20	1	0	0.02	1	0
Substituted benzene	Chlorothalonil	0.01	0.01	2.00	2	0	0.10	2	0
Strobin	Azoxystrobin	0.01	0.01	1.00	1	0	0.20	1	0
Unclassified	Fluopicolide	0.01	0.03	1.00	3	0	0.05	3	0
Herbicide									
Sulfonylurea	Thifensulfuron	0.01	0.01	0.05	1	0	–	1	0
Tomatoes									
Insecticides									
Organochlorine	Dicofol	0.060	0.080	0.02	0	2	0.10	2	0
	Alpha-endosulfan	0.030	0.120	–	7	3	0.002	0	10
	Beta-endosulfan	0.020	0.620	–	8	2	0.002	0	10
	Endosulfan sulfate	0.008	0.080	0.05	8	2	0.002	0	10
Neonicotinoid	Acetamiprid	0.020	0.180	0.20	4	0	0.30	4	0
	Thiamethoxam	0.020	0.020	0.20	1	0	0.20	1	0
Organophosphorus	Chlorpyrifos ethyl	0.010	0.010	0.50	1	0	0.005	0	1
	Triazophos	0.010	0.010	0.01	0	1	–	0	1
Pyrethroid	Alpha-cypermethrin	0.040	0.100	0.50	3	0	0.20	3	0
	Bifenthrin	0.020	0.020	0.30	1	0	0.40	1	0
	Cyfluthrin	0.030	0.030	0.02	0	1	0.002	0	1
	Lambda-cyhalothrin	0.020	0.250	0.10	3	1	0.01	0	4
*N*-Methyl carbamate	Propoxur	0.030	0.030	0.05	2	0	–	0	1
Unclassified	Buprofezin	0.170	0.170	1.00	1	0	0.20	1	0
	Etoxazole	0.020	0.050	0.10	2	0	0.02 (CA)	1	1
	Pyridaben	0.050	0.050	0.30	1	0	0.20	1	0
Fungicides									
Azole	Tebuconazole	0.020	0.020	0.90	2	0	0.20	0	2
	Triadimefon	0.010	0.040	–	3	0	0.50	3	0
	Triadimenol	0.010	0.020	–	2	0	0.02	2	0
	Flusilazole	0.100	0.300	0.02	0	2	–	2	0
	Prochloraz	0.020	0.020	0.05	1	0	–	1	0
Anilide	Boscalid	0.015	0.015	3.00	1	0	3.00 (CA)	1	0
Dicarboximide	Iprodione	0.030	0.030	5.00	1	0	5.00 (CA)	1	0
Substituted benzene	Chlorothalonil	0.050	0.060	2.00	2	0	0.15	2	0
Strobin	Azoxystrobin	0.020	0.020	3.00	3	0	0.20	3	0
Pyrimidine	Pyrimethanil	0.070	0.100	1.00	2	0	0.70 (CA)	2	0
Xylylalanine	Metalaxyl	0.050	0.150	0.50	2	0	0.50	2	0

Among the samples with residues, 25 % (25) of the samples contained pesticide residues below the CU-MRLs whereas 28 % (23) displayed values above safety limits (CU-MRLs). With respect to the detected pesticides in tomatoes, 26 compounds were detected 73 times, of which 14 and 40 exceeded the EU-MRL and Custom Union (CU)-MRL, respectively. In cucumbers, 17 compounds were detected 34 times; 6 and 17 were above the EU-MRL and CU-MRL, respectively. Generally, the EU-MRLs are higher than the more restricted CU-MRLs. Comparing the MRLs, the identical EU-MRL and CU-MRL values include only 0.02 mg kg^−1^ dimethoate and 0.5 mg kg^−1^ metalaxyl. The highest difference between the values of the EU-MRL and CU-MRL is 100-fold difference (0.5 and 0.005 mg kg^−1^) for chlorpyrifos ethyl (Table 2).

The distribution of pesticide residues in samples during the analysed period is presented in Fig. 2. Twenty-nine pesticide residues were detected in the tomatoes and cucumbers; these residues were classified into three groups: (1) the insecticides included organochlorines (endosulfan sulfate, beta and alpha, and dicofol), neonicotinoids (acetamiprid and thiamethoxam), pyrethroids (lambda-cyhalothrin, alpha-cypermethrin, cyfluthrin and bifenthrin), organophosphorus (triazophos, chlorpyrifos ethyl and dimethoate), *N*-methyl carbamate (propoxur) and unclassified (etoxazole, pyridaben and buprofezin); (2) the fungicides included azoles (triadimefon, tebuconazole, triadimenol, flusilazole and prochloraz), substituted benzenes (chlorothalonil), pyrimidines (pyrimethanil), xylylalanines (metalaxyl), dicarboximides (iprodione), anilides (boscalid) and unclassified (fluopicolide); and (3) the herbicides included sulfonylurea (thifensulfuron). The insecticides (16 active substances, 72 detections) were more frequently detected than fungicides (12 active substances, 31 detections) and the sole herbicide.

**Fig. 3:**
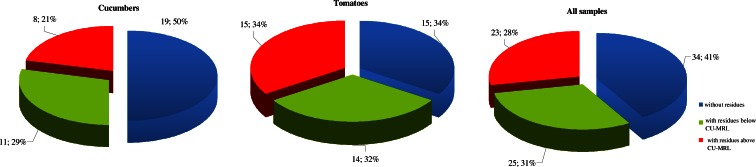
Percent of cucumber and tomato samples with no detectable residues, with residues below and above MRL

When we compare the percentage of samples with pesticide residues depending on the type of vegetables, tomatoes display a higher percentage; only 34 % of the samples do not contain residues and more than one third (34 %) exceed the permitted limits. For cucumbers, half of the samples do not contain residues and approximately 21 % contain residues above the CU-MRL (Fig. 3). Comparing the results obtained in this work with those found in tomato and cucumber samples from other studies (Salghi et al. 2012; Bempah et al. 2011; Latif et al. 2011; Osman et al. 2011; Seddik et al. 2013), the pesticide residues in tomatoes and cucumbers in Kazakhstan present higher amounts and more active substances than those reported from other countries.

Among the most detected pesticides in this study were organochlorine insecticides (15 samples, 41 detections) (Fig. 2). The accumulation of organochlorine compounds (OCCs) in food (Guler et al. 2010) is still a matter of major concern, although the use of most OCCs has been banned or restricted in the majority of countries because of uncertainty surrounding the adverse effects that those residues may have after a lengthy exposure at low doses. The distribution of organochlorine pesticides (OCPs) has been reported by authors in different types of samples (Senthil-Kumar et al. 2001). These organochlorines were detected in tomato and cucumber samples from Morocco (Salghi et al. 2012), Ghana (Bempah et al. 2011), Pakistan (Latif et al. 2011) and Saudi Arabia (Osman et al. 2011). This detection likely reflects the usage pattern of these compounds, which are highly persistent, effective and cheap. Over 60 % of the total organochlorine contamination results from DDT components. The first study of pesticide residues in Kazakhstan (Lozowicka et al. 2013) showed that banned pesticides, such as DDTs, gamma-HCH and aldrin, were found in cereal grain. In the case of endosulfan, the usage of this pesticide in agriculture has been banned in Kazakhstan since 1983. However, nothing is known about its illegal use. The results showed that endosulfan was detected in 13 samples (20.3 %; 10 tomatoes and 3 cucumbers). The concentration was between 0.004 and 0.12 mg kg^−1^ for alpha-isomers, between 0.001 and 0.62 mg kg^−1^ for beta-endosulfan and between 0.003 and 0.08 mg kg^−1^ for endosulfan sulfate. The average concentration for the isomers and sulfate endosulfan was 0.159 mg kg^−1^, and the highest concentration expressed as the sum for an individual tomato sample was 0.88 mg kg^−1^. Endosulfan became a highly controversial agrochemical because of its acute toxicity, potential for bioaccumulation and role as an endocrine disruptor. More than 80 countries (the European Union, Australia, New Zealand, several West African nations, the USA, Brazil and Canada) had already banned the chemical or announced phaseouts by the time the Stockholm Convention ban was agreed upon. Endosulfan is still used extensively in India, China (Shi 2006; Jia et al. 2009) and a few other countries (USA). Endosulfan is a broad-spectrum, non-systemic insecticide and acaricide that is extremely toxic to fish and aquatic invertebrate and has been implicated increasingly in mammalian gonadal toxicity (Saiyed et al. 2003), genotoxicity and neurotoxicity (Silva and Gammon 2009). Endosulfan is also moderately persistent in the soil environment (Jayashree and Vasudevan 2007). Among the OCPs chemically related to DDT, dicofol was detected in tomato samples at concentrations of 0.08 and 0.06 mg kg^−1^. Dicofol is an acaricide used to control many phytophagous mite species, notably the red spider mite, on a range of foods and ornamental crops. Dicofol is still produced and used in China (Liu et al. 2013). Exposure to dicofol can cause adverse health effects and poisoning; the chemical is a possible human carcinogen, and the ADI has been set as 0.002 mg kg^−1^ day^−1^.

Acetamiprid belongs to a new, widely used class of pesticide, the neonicotinoids, and was detected in eight samples (18.7 %). A mean sum value of 0.104 mg kg^−1^ was achieved, with a range of 0.01–0.25 mg kg^−1^. With a similar chemical structure to nicotine, neonicotinoids also share agonist activity at nicotinic acetylcholine receptors (nAChRs). Acetamiprid degrades rapidly by aerobic soil metabolism and has been classified as an *unlikely* human carcinogen. Recently, acetamiprid residues in the environment have received considerable attention because of their potential toxicity to humans (Sanyal et al. 2008). Thiamethoxam also belongs to the neonicotinoids and was detected in one tomato sample and one cucumber sample with a concentration of 0.01 mg kg^−1^, collected in December 2014. Thiamethoxam was developed for foliar/soil applications and used as a seed treatment for most agricultural crops (Vieira et al.^2014^).

In recent decades, pyrethroids have increasingly replaced organochlorine pesticides because of their relatively lower mammalian toxicity, selective insecticide activity and lower environmental persistence. Although posing minimal threat to mammals and avian species, pyrethroids are extremely toxic to aquatic organisms including fish such as the bluegill and lake trout (Saha and Kaviraj 2008). In the tomatoes, the levels of three pyrethroid residues (10 samples, 17.8 %) were 0.02–0.25 mg kg^−1^ for lambda-cyhalothrin, 0.03 mg kg^−1^ for cyfluthrin and 0.1–0.09 mg kg^−1^ for cypermethrin. Cypermethrin is a pyrethroid classified as a moderately toxic chemical (Macedo et al. 2009). In China, cypermethrin is one of the most potent insecticides widely used to control numerous insect pests on fruits, vegetables and field crops. Cypermethrin poses a substantial threat to fish and other aquatic organisms and is highly toxic to honeybees (Lozowicka 2013). Although the effects on humans are still unclear, the US Environmental Protection Agency (EPA) has classified select members (cypermethrin, permethrin and bifenthrin) as possible human carcinogens.

Organophosphorus insecticides (OPIs) were detected in seven samples. Among these insecticides, four samples displayed chlorpyrifos ethyl concentrations above the CU-MRL (one cucumber sample, 0.07 mg kg^−1^, and three tomato samples, 0.01 mg kg^−1^). Chlorpyrifos ethyl has a broad-spectrum activity. Poisoning with this compound can affect the central nervous system, cardiovascular system and respiratory system (Nolan et al. 1984). The estimated risk related to chronic expose for humans to residues of chlorpyrifos by means of a reference dose (RD) of cholinesterase (ChE) is low and amounts to 0.03 mg kg^−1^ b.w. day^−1^. This value considers an uncertainty factor related to the higher sensibility of organisms with not fully developed protection mechanisms, as calculated by the US EPA (0.003 mg kg^−1^ b.w. day^−1^) (IRIS 2007). Tian et al. (2005) suggested that chlorpyrifos displays teratogenic and toxic effects on the mouse embryo in doses lower than those assessed in previous research performed on rats. The effect of chlorpyrifos on human and animal safety remains a current problem to be investigated by the European Commission and US EPA (http://www.tga.gov.au).

The next organophosphorus insecticide, dimethoate, was detected in two samples of cucumbers (0.05 and 0.13 mg kg^−1^, above the CU-MRL, sample no. 2, Table 1). This pesticide is widely used in Europe and in other parts of the world to kill a broad range of insects such as thrips, aphids, mites and whiteflies. Similar to all OPIs, dimethoate acts by interfering with the activities of cholinesterase, an enzyme essential for the proper functioning of the nervous system of insects and humans. Dimethoate is highly toxic to birds and honeybees (Lozowicka 2013) and moderately toxic to most aquatic species and earthworms. Dimethoate is a suspected human teratogen that may affect the reproduction system and is a possible human carcinogen (Usha Rani et al. 1980). The third detected OPI was triazophos (0.01 mg kg^−1^). This pesticide is not registered in Kazakhstan and European Union.

The most frequently detected chemical group among the fungicides was the azole group that included five active substances: triadimefon and tebuconazole (each in four samples), triadimenol (three samples), flusilazole (two samples) and prochloraz (one sample). Azole fungicides are broad-spectrum antifungal compounds used in agriculture. The mechanism of the antifungal action relies on the inhibition of CYP51, resulting in the inhibition of fungal cell growth. Known adverse health effects of azole fungicides are mainly linked to CYP inhibition. Additionally, azole fungicide-induced neurotoxicity has been reported, although the underlying mechanisms are largely unknown (Akoto et al. 2013).

The most frequently detected fungicides were chlorothalonil and azoxystrobin (each in four samples). The levels of chlorothalonil detected in the cucumbers (two samples) and tomatoes (two samples) were between 0.01 and 0.06 mg kg^−1^, whereas the levels of the next fungicide, azoxystrobin, varied between 0.01 and 0.02 mg kg^−1^ in the cucumbers. Fluopicolide, a chemical not registered in Kazakhstan, was detected in three samples (0.01–0.03 mg kg^−1^). Fluopicolide is a mesosystemic fungicide; it translocates toward the stem tips via the xylem but does not translocate toward the roots. Fluopicolide controls a wide range of *Oomycete* (Phycomycete) diseases, late blight (*Phytophthora*) and select *Pythium* species. The mode of action of fluopicolide has not been determined; however, it is a mode of action unlike the known modes of action of other registered fungicides (Sahoo et al. 2014). Pyrimethanil (the pyrimidine group) is not registered in Kazakhstan and was detected in two samples of tomatoes in concentrations of 0.07 and 0.1 mg kg^−1^. Pyrimethanil prevents diseases caused by a wide spectrum of fungi including *Alternaria* spp., *Botrytis cinerea*, *Cercospora* spp., *Cladosporium* spp., *Colletotrichum* spp., *Monilia* spp., *Mycosphaerella* spp., *Penicillium* spp. and *Venturia* spp.

One herbicide was detected in cucumber samples. Thifensulfuron-methyl is a selective systemic herbicide that is absorbed by the leaves and roots of plants and interferes with the synthesis of branched amino acids by the acetolactate synthase (ALS) in sensitive plants. Thifensulfuron-methyl is registered in Kazakhstan but is used for the post-emergence control of broad-leaved weeds in autumn- and spring-sown cereals.

Comparing the results of the 2007 and 2010 European Union-coordinated control programs (EFSA 2013) for tomato samples, the percentage of samples without detectable residues decreased from 68 % in 2007 to 51 % in 2010. The percentage of tomato samples exceeding the MRLs increased from 0.9 % in 2007 to 1.2 % in 2010. However, these results are lower than the detected residues in Kazakh tomatoes.

The fresh cucumbers and tomatoes studied in this research are produced and consumed locally with no or minimal preparation, constituting an important potential source of pesticide residues. Washing under tap water is the most common preparation of these vegetables before consumption (Mehraban et al. 2013). The analytical study of Kazakh tomato and cucumber samples confirmed the presence of non-prohibited use of pesticides in greenhouses, the occurrence of above-permitted concentrations and multiple residue samples. Of the eight permitted insecticides for use in greenhouses of tomatoes and cucumbers, lambda-cyhalothrin, cypermethrin and thiamethoxam were detected; the remaining 13 detected pesticides are not authorised for use. Among the nine permitted fungicides, only three were detected; the remaining nine detected fungicides are not registered in Kazakhstan.

Pesticides, such as endosulfan, dicofol and triazophos, cause the most negative effects on human health and disturb the environment; therefore, pesticides should be restricted. Another difficulty with the uncontrolled use of pesticides is the induction of pest resistance. The intense use of pesticides to kill resistant pests induces additional resistance until further increases in pesticide use actually reduce the agricultural yield. This effect may result in the loss of crops from this region. Integrated pest management encourages the use of fewer pesticide applications and more environment-friendly methods of pest control. Protecting the natural enemies of pests can reduce the pesticide use and increase the productivity.

#### Samples with multi residues

**Fig. 4:**
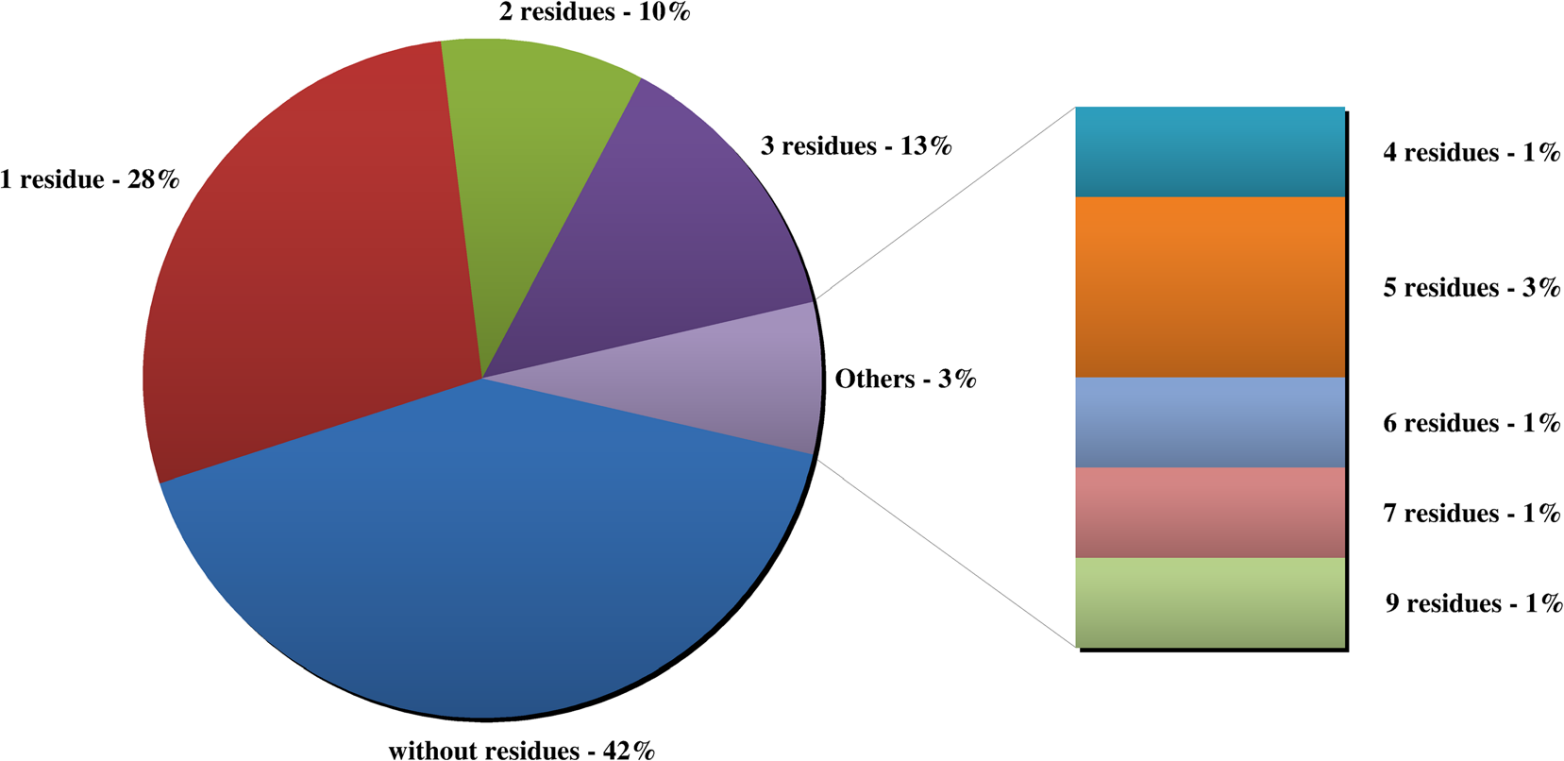
Multiresidue samples

**Fig. 5:**
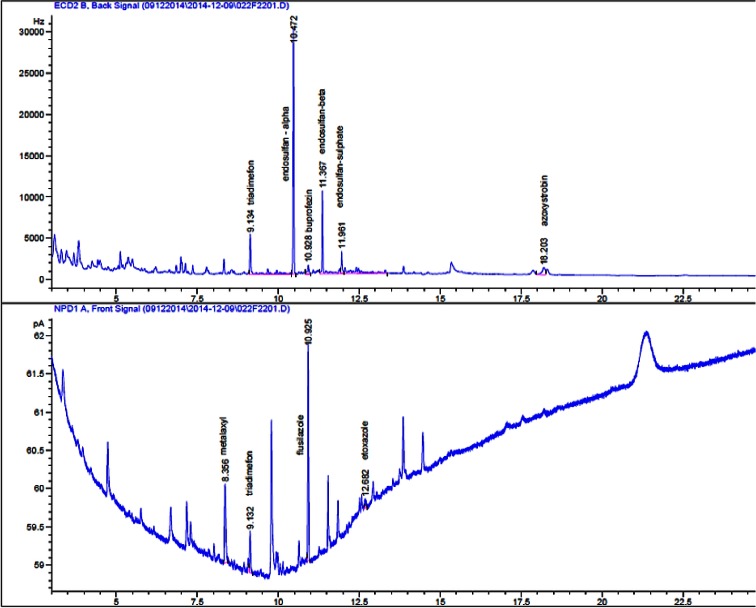
Chromatogram of sample with nine residues (tomato)

Tomatoes and cucumbers are highly sensitive to pests and may therefore require multiple successive applications of pesticide and, consequently, may contained more than one residue. Among the tested vegetables, samples containing one substance (29 %) and multiple active substances (30 %; from two to nine residues) were noted (Fig. 4). Those multiple residues were found most frequently in tomatoes. The most commonly detected residues were a combination of two (acetamiprid and chlorothalonil, endosulfan and tebuconazole, and metalaxyl and chlorothalonil) and three pesticides (acetamiprid, chlorothalonil and fluopicolide, and triadimefon, triadimenol and tebuconazole) (23 %). Six pesticide residues, including the alpha, beta and sulfate of endosulfan; acetamiprid; lambda-cyhalothrin; and tebuconazole, were detected in one sample. One sample of tomato contained nine pesticide residues, and among them, four fungicides (azoxystrobin, metalaxyl, flusilazole and triadimefon) had a range of 0.02–0.15 mg kg^−1^ and three insecticides (endosulfan (sum of 0.06 mg kg^−1^), buprofezin and etoxazole) had a mean concentration of 0.6 mg kg^−1^. These samples with multiresidue pesticides carry a higher risk to the health of consumers (Fig. 5).

### Risk of exposure

The acute (short-term) and chronic (long-term) health effects of pesticides warrant attention and concern. Whereas the acute toxicity of most pesticides is well documented (Ecobichon et al. 1990), information on chronic human illnesses, such as cancer, is not as sound. The WHO estimates that food sources contribute approximately 80 % of the daily intake of a particular contaminant. Among the pesticides detected in this study, 1 carcinogen, 16 possible carcinogens, 3 mutagens and endocrine disrupters, 11 suspected endocrine disruptors, 6 reproductive toxins, 8 suspected developmental toxins, 9 neurotoxins, 8 respiratory irritants and 11 skin and eye irritants were noted (http://sitem.herts.ac.uk/aeru/ppdb/en/573.htm) (Table 2).

The risk from pesticide residues in tomatoes and cucumbers was evaluated on the basis of two toxicological limit values: the ADI and the ARfD. In this study, the assessment of chronic and acute health risk of consumers connected with the consumption of vegetables from Kazakhstan containing pesticide residues at the average and highest levels was conducted on the basis of available epidemiological studies performed for the WHO and EFSA diets (because of a lack of full studies performed for Kazakh consumers) (Table 3). The Global Environment Monitoring System (GEMS)/Food consumption areas are based on the geographic proximity between 183 countries. Kazakhstan belongs to cluster D (Europe/Middle East). This cluster included 20 countries: Armenia, Azerbaijan, Georgia, Kazakhstan, Kyrgyzstan, Russian Federation, Tajikistan, Turkmenistan, Ukraine and Uzbekistan, among others. The southern parts of Europe and Asia, such as Greece, Israel, Italy, Spain, Turkey and the United Arab Emirates, have been classified into identical consumption cluster diet B (GEMS/Food 2012). The GEMS/Food cluster diets are based on similarities between dietary patterns, and Kazakhstan belongs to G02: Albania, Bosnia and Herzegovina, Georgia, Kazakhstan, Kyrgyzstan, Republic of Moldova, Republic of Montenegro and Ukraine. In the diet of Kazakh people (region G02), fruiting vegetables (like tomatoes and cucumbers) consist of approximately 10 % (142.6 g day^−1^) of the daily food intake (two total diet of 121 g day^−1^).

**Table 3:** Acute risk assessment (T - tomatoes, C - cucumbers)

Group of pesticide	Active substance (toxicity profile)	ARfD (mg kg^−1^ b.w. day^−1^)	Highest residue level (mg kg^−1^)	aHI (%)					
				WHO cluster diet B	WHO cluster diet D	Italian kid/toddler	Italian adult	Denmark child	Denmark adult
Neonicotinoid	Acetamiprid (EI, SI)	0.10	0.15 (C)	0.22	0.20	0.03	0.02	1.72	0.28
			0.02 (T)	0.43	0.14	0.20	0.16	0.07	0.06
	Thiamethoxam (PC)	0.50	0.01 (C)	0.00	0.00	0.00	0.00	0.02	0.00
			0.02 (T)	0.09	0.03	0.04	0.03	0.01	0.01
Organophosphorus	Chlorpyrifos ethyl (EI, N, RT, SED, SI)	0.10	0.07 (C)	0.10	0.09	0.01	0.01	0.80	0.13
			0.01 (T)	0.22	0.07	0.10	0.08	0.04	0.03
	Dimethoate (PC, SED)	0.01	0.13 (C)	1.93	1.76	0.24	0.20	14.89	2.45
	Triazophos (EI, SI)	0.001	0.10 (T)	215.83	70.82	99.76	81.46	37.23	28.95
Organochlorine	Alpha-endosulfan (M, N, PC, SED)	0.02	0.04 (C)	0.30	0.27	0.04	0.03	2.29	0.38
			0.12 (T)	12.95	4.25	5.99	4.89	2.23	1.74
	Beta-endosulfan (M, N, PC, SED)	0.02	0.02 (C)	0.15	0.14	0.02	0.02	1.15	0.19
			0.62 (T)	66.91	21.95	30.93	25.25	11.54	8.97
	Endosulfan sulfate (M, N, PC, SED)	0.02	0.02 (C)	0.15	0.14	0.02	0.02	1.15	0.19
			0.08 (T)	8.63	2.83	3.99	3.26	1.49	1.16
Azole	Prochloraz (PC, RT, SED)	0.025	0.02 (T)	1.73	0.57	0.80	0.65	0.30	0.23
	Flusilazole (PC, RT)	0.005	0.3 (T)	129.50	42.49	59.86	48.88	22.34	17.37
	Tebuconazole (PC, RT)	0.03	0.25 (C)	1.23	1.13	0.16	0.13	9.55	1.57
			0.02 (T)	1.44	0.47	0.67	0.54	0.25	0.19
	Triadimefon (PC, RT, SED)	0.08	0.02 (C)	0.04	0.03	0.00	0.00	0.29	0.05
			0.04 (T)	1.08	0.35	0.50	0.41	0.19	0.14
	Triadimenol (ED, RT)	0.05	0.02 (C)	0.06	0.05	0.01	0.01	0.46	0.08
			0.02 (T)	0.86	0.28	0.40	0.33	0.15	0.12
Pyrethroid	Lambda-cyhalothrin (ED, EI, SI)	0.0075	0.02 (C)	0.40	0.36	0.05	0.04	3.05	0.50
			0.25 (T)	71.94	23.61	33.25	27.15	12.41	9.65
	Alpha-cypermethrin (ED, EI, PC, SED SI)	0.20	0.10 (T)	1.08	0.35	0.50	0.41	0.19	0.14
	Bifenthrin (ED, PC)	0.03	0.02 (T)	1.44	0.47	0.67	0.54	0.25	0.19
	Cyfluthrin (N)	0.02	0.03 (T)	3.24	1.06	1.50	1.22	0.56	0.43
Substituted benzene	Chlorothalonil (PC)	0.60	0.01 (C)	0.00	0.00	0.00	0.00	0.02	0.00
Unclassified	Fluopicolide (PC)	0.18	0.03 (C)	0.02	0.02	0.00	0.00	0.19	0.03
	Buprofezin (PC)	0.50	0.17 (T)	0.73	0.24	0.34	0.28	0.13	0.10
	Pyridaben (EI, SI)	0.05	0.05 (T)	2.16	0.71	1.00	0.81	0.37	0.29
Xylylalanine	Metalaxyl (EI, SI)	0.50	0.15 (T)	0.65	0.21	0.30	0.24	0.11	0.09

*T* tomatoes, *C* cucumbers, *ED* endocrine disruptor, *EI* eye irritant, *M* mutagen, *N* neurotoxicant, *PC* possible carcinogen, *RT* reproduction/development effects, *SI* skin irritant, *SED* suspected endocrine disruptor

During the assessment of the long-term consumer risk, the study assumed a cautious approach using conservative guidelines, which inflated the risk. Based on the results (Table 1), the chronic intakes of the 29 pesticide residues are rather low compared to the ADI. The safety of Kazakh consumers (cluster D, adults and children) thus seems to be generally under control in terms of pesticide intake through the consumption of tomatoes and cucumbers. The HQ was calculated for both pesticides and commodities. For select residues, such as buprofezin, dicofol, dimethoate, flusilazole, lambda-cyhalothrin and triazophos, the HQ for the high consumer (97.5th percentile) was 2.05 , 4.22 , 10.85 , 12.05 , 1.74 and 1.21 % of the ADI, respectively (for cluster D). In the case of groups of pesticides, a cumulative risk should be considered because these compounds may have a common mechanism (e.g. organophosphorus displays an acetylcholinesterase inhibition). The HQs were summed, and the chronic hazard index (cHI) for select chemical groups is as follows: 6.89 % for organochlorines (endosulfan and dicofol), 12.57 % for organophosphorus (triazophos, chlorpyrifos and dimethoate) and 12.73 % for fungicidal azoles (triadimefon, tebuconazole, triadimenol, flusilazole and prochloraz). With respect to children, the ADI was below cHI = 50 %. The results show a risk associated with exposure via tomato and cucumber consumption, and a special precaution should be taken with the possible aggregate exposure to these chemicals from multiple sources of nutrition and the domestic use of pesticides.

The deterministic acute exposure was calculated only for compounds exceeding the MRLs for both commodities, and it is expressed as an acute hazard index (aHI) for clusters D and B, adults and children based on the highest consumption at the 97.5th percentile and the highest concentrations of pesticide residues detected in tomatoes and cucumbers (Table 3). To evaluate whether an observed violation of an MRL can lead to a risk to the consumer, the actual risk to the most critical consumer group must be estimated. Generally, children from 1.5 to 6 years of age are considered as the most vulnerable group because they tend to eat a large number of single units of one food commodity in 1 day. The samples with pesticide residues exceeding the MRLs do not constitute a real threat to health. In our study, the highest risk associated with the consumption of cucumbers containing lambda-cyhalothrin could occur among consumers in cluster B. The acute risk from endosulfan, flusilazole, lambda-cyhalothrin and triazophos in tomatoes was the highest, and the aHI values are 29.03 , 42.49 , 70.82 and 23.61 % of the ARfD for consumers in cluster D, respectively, and 88.49 , 129.50 71.94 and 215.83 % of the ARfD for consumers in cluster B, respectively. The evaluation of the consumer health risk connected with the contamination of vegetables with pesticide residues shows combinations for which a critical intake situation could not be excluded. Therefore, risk management activities have already been put into effect by withdrawing authorizations or lowering the MRLs. The dietary pesticide intakes estimated in this study considered only exposures from two types of vegetables and did not include other food products such as grains, dairy, fish and meats. Therefore, this value is an underestimation of the total exposure to pesticides. Nevertheless, pesticide residue monitoring programs are increasingly important and essential to ensure minimal residue levels in food (Lozowicka 2015; Lozowicka et al. 2014; Yuan et al. 2014; Wu et al. 2014).

## Conclusion

The results of this study support our hypothesis about the presence of pesticides in greenhouse tomatoes and cucumbers sold in market outlets and supermarkets in Kazakhstan. Tomato and cucumber samples contain pesticide residues in more than half of all the samples. Multiple samples exceeded the MRLs, containing residues and metabolites of illegal pesticides. Thus, the appropriate pesticide use and a residue monitoring system must be established in Kazakhstan. Strict penalties against local greenhouse producers violating rules of pesticide use must be arranged. Producers must submit for pesticide residue testing of their farm outputs at accredited toxicology labs with modern equipment and elaborate detection methods. A centralised network of pesticide toxicology labs must be organised at the entry points of the state borders. Retailers must possess a certificate of pesticide residue analysis and the origin of marketed vegetables. Nationwide pesticide reduction programs and biological pest control measures should be promoted and introduced into greenhouse vegetable production to protect the health of consumers in Kazakhstan. The performed risk assessment showed that the pesticide residues detected in vegetables will not constitute a risk for Kazakh people from cluster D. However, the dietary pesticide exposures estimated in this study considered only exposures from tomatoes and cucumbers and did not include other food products such as fruits, other vegetables, grains, dairy, fish and meats.

## Electronic supplementary material

ESM 1 (DOC 532 kb)

ESM 2 (RTF 271 kb)
