# Supplementary material for: Studies of pesticide residues in tomatoes and cucumbers from Kazakhstan and the associated health risks
Source: Environ Monit Assess. 2015 Sep 4;187(10):609. doi: 10.1007/s10661-015-4818-6 (PMC4559566; doi:10.1007/s10661-015-4818-6)
Supplement: Supplementary file 1 — (DOC 532 kb) [file 10661_2015_4818_MOESM1_ESM.doc]

**Table S1** Recoveries, repeatability (RSD), limit of quantification (LOQ), and determination (LOD) for 184 pesticides validated on tomatoes and cucumbers

| L.p. | Mode of action | Pesticide | R^2^ | 1^st^ fort. level  (mg kg^-1^) | Mean recovery ± RSD (n=3)  (%) | 2^nd^ fort. level  (mg kg^-1^) | Mean recovery ± RSD (n=3)  (%) | 3^rd^ fort. level  (mg kg^-1^) | Mean recovery ± RSD (n=3)  (%) | LOD  (mg/kg) | LOQ  (mg kg^-1^) |
| --- | --- | --- | --- | --- | --- | --- | --- | --- | --- | --- | --- |
|  | FUNGICIDE | azaconazole | 0.99894 | 0.010 | 107.8 ± 2.2 | 0.100 | 94.9 ± 5.7 | 0.500 | 104.6 ± 6.8 | 0.003 | 0.010 |
|  |  | azoxystrobin | 0.99997 | 0.010 | 90.9 ± 3.3 | 0.100 | 90.1 ± 8.4 | 0.500 | 108.6 ± 5.3 | 0.002 | 0.005 |
|  |  | benalaxyl | 0.99580 | 0.010 | 95.0 ± 3.0 | 0.100 | 93.4 ± 0.9 | 0.500 | 104.6 ± 6.2 | 0.024 | 0.080 |
|  |  | bitertanol | 0.99597 | 0.010 | 90.1 ± 3.2 | 0.100 | 92.6 ± 10.3 | 0.500 | 106.9 ± 5.0 | 0.003 | 0.010 |
|  |  | boscalid | 0.99963 | 0.020 | 106.5 ± 0.8 | 0.200 | 98.5 ± 4.2 | 1.000 | 105.9 ± 4.5 | 0.006 | 0.020 |
|  |  | bromuconazole | 0.99922 | 0.030 | 104.4 ± 2.1 | 0.300 | 85.6 ± 4.7 | 1.500 | 106.6 ± 8.2 | 0.006 | 0.020 |
|  |  | bupirimate | 0.99984 | 0.010 | 96.9 ± 2.3 | 0.100 | 76.5 ± 6.7 | 0.500 | 110.9 ± 7.3 | 0.003 | 0.010 |
|  |  | captan | 0.99711 | 0.030 | 85.4 ± 4.0 | 0.300 | 89.1 ± 3.4 | 1.500 | 109.9 ±2.8 | 0.003 | 0.010 |
|  |  | chlorothalonil | 0.99821 | 0.010 | 106.1 ± 3.8 | 0.100 | 97.9 ± 0.9 | 0.500 | 99.3 ± 6.1 | 0.002 | 0.005 |
|  |  | cyazofamid | 0.99739 | 0.010 | *52.8 ± 2.4* | 0.100 | 64.2 ± 6.8 | 0.500 | 69.3 ± 7.0 | 0.002 | 0.005 |
|  |  | cyproconazole | 0.99839 | 0.020 | 113.5 ± 2.0 | 0.200 | 97.0 ± 3.1 | 1.000 | 102.9 ± 5.1 | 0.003 | 0.010 |
|  |  | cyprodinil | 0.99964 | 0.004 | 93.4 ± 4.5 | 0.040 | 93.4 ± 10.2 | 0.200 | 94.6 ± 7.3 | 0.003 | 0.010 |
|  |  | dichlofluanid | 0.99995 | 0.006 | 98.9 ± 1.6 | 0.060 | 78.9. ± 7.2 | 0.300 | 107.1 ± 8.3 | 0.003 | 0.010 |
|  |  | dicloran | 0.99769 | 0.007 | 98.5 ± 3.3 | 0.070 | 85.9 ± 9.4 | 0.350 | 105.9 ± 6.2 | 0.003 | 0.010 |
|  |  | difenoconazole | 0.99711 | 0.010 | 90.0 ± 2.2 | 0.100 | 95.1 ± 3.6 | 0.500 | 106.5 ± 9.3 | 0.002 | 0.005 |
|  |  | dimetomorph | 0.99858 | 0.010 | 106.8 ± 2.9 | 0.100 | 97.1 ± 5.9 | 0.500 | 115.9 ± 8.0 | 0.002 | 0.005 |
|  |  | dimoxystrobin | 0.99987 | 0.003 | 96.5 ± 0.5 | 0.300 | 91.2 ± 8.2 | 1.500 | 104.6 ± 5.3 | 0.003 | 0.010 |
|  |  | diniconazole | 0.99984 | 0.010 | 96.9 ± 2.3 | 0.100 | 76.5 ± 6.7 | 0.500 | 110.9 ± 7.3 | 0.003 | 0.010 |
|  |  | diphenylamine | 0.99971 | 0.010 | 88.7 ± 2.4 | 0.040 | 90.1 ± 8.2 | 0.200 | 108.9 ± 4.7 | 0.006 | 0.020 |
|  |  | epoxiconazole | 0.99991 | 0.010 | 103.7 ± 4.0 | 0.100 | 70.7 ± 4.5 | 0.500 | 87.2 ± 6.8 | 0.002 | 0.005 |
|  |  | famoksadon | 0.99839 | 0.020 | 113.5 ± 2.0 | 0.200 | 97.0 ± 3.1 | 1.000 | 102.9 ± 5.1 | 0.003 | 0.010 |
|  |  | fenarimol | 0.99750 | 0.010 | 88.4 ± 0.7 | 0.100 | 94.5 ± 5.3 | 0.500 | 95.3 ± 8.9 | 0.002 | 0.005 |
|  |  | fenbuconazole | 0.99991 | 0.010 | *54.6 ± 3.5* | 0.100 | *43.8 ± 8.6* | 0.500 | *55.2 ± 9.7* | 0.003 | 0.010 |
|  |  | fenchlorfos | 0.99964 | 0.004 | 93.4 ± 4.5 | 0.040 | 93.4 ± 10.2 | 0.200 | 94.6 ± 7.3 | 0.003 | 0.010 |
|  |  | fenhexamid | 0.99997 | 0.010 | 88.4 ± 0.6 | 0.100 | 88.3 ± 8.5 | 0.500 | 107.6 ± 7.6 | 0.002 | 0.005 |
|  |  | fenpropimorph | 0.99960 | 0.002 | 112.2 ± 4.5 | 0.020 | 93.1 ± 5.4 | 0.100 | 106.6 ± 8.0 | 0.003 | 0.010 |
|  |  | fludioxonil | 0.99852 | 0.010 | 101.7 ± 1.5 | 0.100 | 76.1 ± 0.5 | 0.500 | 105.7 ± 8.3 | 0.002 | 0.005 |
|  |  | fluopicolide | 0.99850 | 0.010 | 93.1 ± 3.6 | 0.300 | 79.4 ± 6.7 | 1.500 | 108.6 ± 5.6 | 0.003 | 0.010 |
|  |  | flusilazole | 0.99994 | 0.010 | 113.0 ± 6.7 | 0.100 | 93.1 ± 9.4 | 0.500 | 103.8 ± 8.2 | 0.003 | 0.010 |
|  |  | flutriafol | 0.99893 | 0.003 | 97.2 ± 1.5 | 0.300 | 85.2 ± 2.9 | 1.500 | 91.6 ± 6.2 | 0.001 | 0.003 |
|  |  | folpet | 0.99978 | 0.010 | 97.4 ± 1.6 | 0.100 | 94.1 ± 10.4 | 0.500 | 109.8 ± 5.3 | 0.003 | 0.010 |
|  |  | hexaconazole | 0.99972 | 0.010 | 102.7 ± 1.6 | 0.100 | 96.6 ± 7.6 | 0.500 | 96.8 ± 8.0 | 0.002 | 0.006 |
|  |  | imazalil | 0.99673 | 0.010 | 108.5 ± 2.3 | 0.100 | 92.1 ± 8.3 | 0.500 | 104.2 ± 8.4 | 0.002 | 0.006 |
|  |  | iprodione | 0.99995 | 0.010 | 92.4 ± 2.5 | 0.100 | 92.5 ± 1.0 | 0.500 | 94.6 ± 9.4 | 0.002 | 0.007 |
|  |  | imibenconazole | 0.99839 | 0.020 | 113.5 ± 2.0 | 0.200 | 97.0 ± 3.1 | 1.000 | 102.9 ± 5.1 | 0.003 | 0.010 |
|  |  | kresoxim-methyl | 0.99865 | 0.010 | 91.7 ± 1.0 | 0.100 | 82.1 ± 3.8 | 0.500 | 97.6 ± 9.6 | 0.001 | 0.004 |
|  |  | mefenoxam | 0.99987 | 0.003 | 96.5 ± 0.5 | 0.300 | 91.2 ± 8.2 | 1.500 | 104.6 ± 5.3 | 0.003 | 0.010 |
|  |  | mepanipyrim | 0.99700 | 0.006 | 95.6 ± 3.4 | 0.060 | 92.5 ± 8.3 | 0.300 | 105.8 ± 7.4 | 0.006 | 0.020 |
|  |  | metalaxyl | 0.99967 | 0.005 | 94.8 ± 1.3 | 0.050 | 77.3 ± 0.5 | 0.250 | 105.9 ± 6.3 | 0.002 | 0.008 |
|  |  | metconazole | 0.99839 | 0.020 | 113.5 ± 2.0 | 0.200 | 97.0 ± 3.1 | 1.000 | 102.9 ± 5.1 | 0.003 | 0.010 |
|  |  | myclobuthanil | 0.99964 | 0.004 | 93.4 ± 4.5 | 0.040 | 93.4 ± 10.2 | 0.200 | 94.6 ± 7.3 | 0.003 | 0.010 |
|  |  | oxadixyl | 0.99920 | 0.005 | 115.1 ± 0.6 | 0.050 | 74.8 ± 5.5 | 0.250 | 99.8 ± 4.5 | 0.002 | 0.006 |
|  |  | paclobutrazol | 0.99798 | 0.010 | 106.8 ± 3.0 | 0.100 | 71.4 ± 8.9 | 0.500 | 77.3 ± 6.0 | 0.002 | 0.008 |
|  |  | penconazole | 0.99983 | 0.020 | 90.7 ± 3.5 | 0.200 | 77.0 ± 2.0 | 1.000 | 108.5 ± 2.5 | 0.006 | 0.020 |
|  |  | pencycuron | 0.99893 | 0.003 | 97.2 ± 1.5 | 0.300 | 85.2 ± 2.9 | 1.500 | 91.6 ± 6.2 | 0.001 | 0.003 |
|  |  | picoxystrobin | 0.99697 | 0.020 | 104.9 ± 4.6 | 0.200 | 92.3 ± 5.9 | 1.000 | 104.2 ± 8.2 | 0.006 | 0.020 |
|  |  | prochloraz | 0.99995 | 0.010 | 87.5 ± 2.5 | 0.100 | 92.5 ± 6.8 | 0.500 | 105.2 ± 5.1 | 0.003 | 0.010 |
|  |  | procymidone | 0.99970 | 0.006 | 96.6 ± 5.4 | 0.060 | 95.1 ± 3.4 | 0.300 | 105.8 ± 6.2 | 0.002 | 0.005 |
|  |  | propiconazole | 0.99528 | 0.020 | 92.8 ± 2.5 | 0.200 | 92.6 ± 7.8 | 1.000 | 99.5 ± 5.1 | 0.001 | 0.003 |
|  |  | pyraclostrobin | 0.99996 | 0.004 | 92.4 ± 2.1 | 0.040 | 78.3 ± 7.1 | 0.200 | 98.6 ± 8.3 | 0.001 | 0.003 |
|  |  | pyrazophos | 0.99795 | 0.005 | 107.2 ± 1.3 | 0.050 | 81.9 ± 8.3 | 0.250 | 100.9 ± 7.2 | 0.001 | 0.002 |
|  |  | pyrimethanil | 0.99840 | 0.008 | 93.9 ± 2.1 | 0.080 | 95.2 ± 6.5 | 0.400 | 98.7 ± 5.3 | 0.001 | 0.004 |
|  |  | quinoxyfen | 0.99987 | 0.003 | 96.5 ± 0.5 | 0.300 | 91.2 ± 8.2 | 1.500 | 104.6 ± 5.3 | 0.003 | 0.010 |
|  |  | quintozene | 0.99893 | 0.003 | 97.2 ± 1.5 | 0.300 | 85.2 ± 2.9 | 1.500 | 91.6 ± 6.2 | 0.001 | 0.003 |
|  |  | tebuconazole | 0.99970 | 0.010 | 108.0 ± 0.8 | 0.100 | 80.3 ± 8.8 | 0.500 | 91.5 ± 9.5 | 0.002 | 0.005 |
|  |  | tecnazene | 0.99976 | 0.030 | 90.9 ± 4.1 | 0.300 | 101.6 ± 1.1 | 1.500 | 109.8 ± 6.5 | 0.002 | 0.005 |
|  |  | tetraconazole | 0.99989 | 0.010 | *113.3* ± 2.8 | 0.100 | *129.6 ± 5.6* | 0.500 | *121.0* ± 3.1 | 0.002 | 0.005 |
|  |  | tolclofos-methyl | 0.99968 | 0.010 | 99.0 ± 4.5 | 0.100 | 87.4 ± 4.5 | 0.500 | 122.1 ± 5.5 | 0.001 | 0.002 |
|  |  | tolylfluanid | 0.99847 | 0.020 | 94.8 ± 3.8 | 0.200 | 90.3 ± 2.3 | 1.000 | 103.8 ± 8.0 | 0.001 | 0.003 |
|  |  | triadimefon | 0.99807 | 0.003 | 94.5 ± 4.4 | 0.030 | 76.3 ± 8.5 | 0.150 | 108.6 ± 9.6 | 0.002 | 0.005 |
|  |  | triadimenol | 0.99726 | 0.005 | 84.8 ± 2.2 | 0.050 | 95.0 ± 2.4 | 0.250 | 106.8 ± 6.1 | 0.003 | 0.010 |
|  |  | trifloxystrobin | 0.99989 | 0.010 | 97.4 ± 1.2 | 0.100 | 90.7 ± 1.9 | 0.500 | 101.9 ± 4.2 | 0.001 | 0.002 |
|  |  | vinclozolin | 0.99919 | 0.004 | 95.9 ± 2.1 | 0.040 | 84.3 ± 2.4 | 0.200 | 99.8 ± 5.4 | 0.002 | 0.005 |
|  |  | zoxamide | 0.99697 | 0.020 | 104.9 ± 4.6 | 0.200 | 92.3 ± 5.9 | 1.000 | 104.2 ± 8.2 | 0.006 | 0.020 |
|  | HERBICIDE | acetochlor | 0.99995 | 0.010 | 87.5 ± 2.5 | 0.100 | 92.5 ± 6.8 | 0.500 | 105.2 ± 5.1 | 0.003 | 0.010 |
|  |  | atrazine | 0.99995 | 0.010 | 86.6 ± 5.0 | 0.200 | 83.4 ± 11.3 | 1.000 | 103.5 ± 3.1 | 0.003 | 0.010 |
|  |  | bromacil | 0.99989 | 0.010 | 106.7 ± 3.3 | 0.100 | 96.7 ± 3.6 | 0.500 | 106.8 ± 7.1 | 0.003 | 0.010 |
|  |  | chlorpropham | 0.99940 | 0.010 | 105.2 ± 3.3 | 0.100 | 82.1 ± 3.8 | 0.500 | 105.9 ± 8.6 | 0.002 | 0.005 |
|  |  | cyanazine | 0.99990 | 0.010 | 94.7 ± 2.5 | 0.100 | 100.2 ± 5.9 | 0.500 | 103.2 ± 9.0 | 0.002 | 0.005 |
|  |  | cyprazine | 0.99723 | 0.010 | 94.7 ± 3.3 | 0.100 | 85.6 ± 2.8 | 0.500 | 116.2 ± 2.4 | 0.002 | 0.005 |
|  |  | diflufenican | 0.99990 | 0.010 | 85.3 ± 3.9 | 0.100 | 95.6 ± 6.6 | 0.500 | 104.2 ± 6.5 | 0.002 | 0.005 |
|  |  | flurochloridine | 0.99283 | 0.010 | 106.3 ± 5.1 | 0.100 | 97.5 ± 5.4 | 0.500 | 100.6 ± 5.3 | 0.002 | 0.005 |
|  |  | lenacil | 0.99745 | 0.005 | 91.2 ± 3.9 | 0.050 | 83.5 ± 1.1 | 0.250 | 92.8 ± 4.9 | 0.006 | 0.020 |
|  |  | metazachlor | 0.99283 | 0.010 | 106.3 ± 5.1 | 0.100 | 97.5 ± 5.4 | 0.500 | 100.6 ± 5.3 | 0.002 | 0.005 |
|  |  | metholachlor | 0.99695 | 0.010 | 100.6 ± 2.4 | 0.100 | 98.6 ± 2.5 | 0.500 | 96.5 ± 8.3 | 0.002 | 0.005 |
|  |  | metribuzin | 0.99995 | 0.010 | 93.7 ± 2.2 | 0.100 | 79.8 ± 9.8 | 0.500 | 119.6 ± 6.7 | 0.002 | 0.005 |
|  |  | napropamide | 0.99948 | 0.010 | 96.5 ± 2.9 | 0.100 | 89.4 ± 5.2 | 0.500 | 106.8 ± 8.1 | 0.002 | 0.005 |
|  |  | nitrofen | 0.99903 | 0.010 | 78.0 ± 0.9 | 0.100 | 100.6 ± 5.8 | 0.500 | 106.7 ± 4.3 | 0.002 | 0.005 |
|  |  | oxyflurofen | 0.99839 | 0.020 | 113.5 ± 2.0 | 0.200 | 97.0 ± 3.1 | 1.000 | 102.9 ± 5.1 | 0.003 | 0.010 |
|  |  | pendimethalin | 0.99984 | 0.010 | 93.7 ± 3.1 | 0.100 | 104.5 ± 9.8 | 0.500 | 103.1 ± 9.2 | 0.003 | 0.010 |
|  |  | prometryn | 0.99991 | 0.010 | 104.7 ± 1.7 | 0.100 | 85.8 ± 2.4 | 0.500 | 104.8 ± 7.2 | 0.003 | 0.010 |
|  |  | propachlor | 0.99490 | 0.020 | 79.6 ± 2.4 | 0.200 | *65.8 ± 3.9* | 1.000 | 105.8 ± 7.3 | 0.003 | 0.010 |
|  |  | propaquizafop | 0.99839 | 0.020 | 113.5 ± 2.0 | 0.200 | 97.0 ± 3.1 | 1.000 | 102.9 ± 5.1 | 0.003 | 0.010 |
|  |  | propazine | 0.99964 | 0.004 | 93.4 ± 4.5 | 0.040 | 93.4 ± 10.2 | 0.200 | 94.6 ± 7.3 | 0.003 | 0.010 |
|  |  | prometrine | 0.99592 | 0.005 | 105.5 ± 2.7 | 0.050 | 96.1 ± 1.5 | 0.250 | 104.2 ± 6.4 | 0.003 | 0.010 |
|  |  | propham | 0.99997 | 0.005 | 93.1 ± 6.2 | 0.050 | 101.0 ± 5.3 | 0.250 | 107.2 ± 5.3 | 0.002 | 0.005 |
|  |  | propyzamide | 0.99930 | 0.020 | 104.0 ± 0.2 | 0.200 | 94.4 ± 7.7 | 1.000 | 118.2 ± 6.4 | 0.003 | 0.010 |
|  |  | simazine | 0.99283 | 0.010 | 106.3 ± 5.1 | 0.100 | 97.5 ± 5.4 | 0.500 | 100.6 ± 5.3 | 0.002 | 0.005 |
|  |  | thifensulfuron | 0.99283 | 0.010 | 99.3 ± 4.1 | 0.100 | 94.5 ± 5.5 | 0.500 | 102.6 ± 3.8 | 0.002 | 0.005 |
|  |  | trifluralin | 0.99783 | 0.010 | 89.4 ± 2.5 | 0.100 | 98.4 ± 4.5 | 0.500 | 115.4 ± 1.2 | 0.003 | 0.010 |
|  | INSECTICIDE | acetamiprid | 0.99994 | 0.010 | 90.3 ± 3.1 | 0.100 | 80.3 ± 3.6 | 0.500 | 105.8 ± 6.4 | 0.003 | 0.010 |
|  |  | acrinathrin | 0.99839 | 0.020 | 113.5 ± 2.0 | 0.200 | 97.0 ± 3.1 | 1.000 | 102.9 ± 5.1 | 0.003 | 0.010 |
|  |  | aldrine | 0.99990 | 0.005 | 103.9 ± 1.5 | 0.050 | 85.6 ± 1.2 | 0.250 | 95.2 ± 8.2 | 0.001 | 0.003 |
|  |  | alpha-cypermethrin | 0.99864 | 0.010 | 98.5 ± 5.3 | 0.100 | 97.4 ± 3.4 | 0.500 | 103.9 ± 8.4 | 0.002 | 0.005 |
|  |  | alpha-endosulfan | 0.99984 | 0.005 | 109.1 ± 5.0 | 0.050 | 90.6 ± 2.5 | 0.250 | 95.8 ± 6.2 | 0.001 | 0.004 |
|  |  | alpha-HCH | 0.99999 | 0.005 | 106.7 ± 2.0 | 0.050 | 90.5 ± 3.5 | 0.250 | 101.6 ± 5.0 | 0.003 | 0.010 |
|  |  | azinphos-ethyl | 0.99730 | 0.010 | 96.1 ± 6.5 | 0.100 | 92.6 ± 4.8 | 0.500 | 104.6 ± 0.5 | 0.002 | 0.005 |
|  |  | azinphos-methyl | 0.99969 | 0.010 | 106.6 ± 0.8 | 0.100 | 97.4 ± 7.6 | 0.500 | 94.6 ± 16.2 | 0.002 | 0.005 |
|  |  | beta-cyfluthrin | 0.99958 | 0.010 | 93.0 ± 1.7 | 0.100 | 95.3 ± 14.2 | 0.500 | 113.7 ± 5.1 | 0.003 | 0.010 |
|  |  | beta-endosulfan | 0.99988 | 0.020 | 104.0 ± 0.4 | 0.200 | *126.8 ± 5.1* | 1.000 | 118.9 ± 5.8 | 0.006 | 0.020 |
|  |  | beta-HCH | 0.99995 | 0.010 | 94.5 ± 2.2 | 0.100 | 95.6 ± 1.8 | 0.500 | 106.5 ± 5.2 | 0.002 | 0.005 |
|  |  | bifenthrin | 0.99894 | 0.010 | 107.8 ± 2.2 | 0.100 | 94.9 ± 5.7 | 0.500 | 104.6 ± 6.8 | 0.003 | 0.010 |
|  |  | bromophos-ethyl | 0.99283 | 0.010 | 106.3 ± 5.1 | 0.100 | 97.5 ± 5.4 | 0.500 | 100.6 ± 5.3 | 0.002 | 0.005 |
|  |  | bromophos-methyl | 0.99987 | 0.003 | 96.5 ± 0.5 | 0.300 | 91.2 ± 8.2 | 1.500 | 104.6 ± 5.3 | 0.003 | 0.010 |
|  |  | bromopropylate | 0.99910 | 0.020 | 105.3 ± 2.7 | 0.200 | 94.8 ± 5.6 | 1.000 | 100.2 ± 6.2 | 0.006 | 0.020 |
|  |  | buprofezin | 0.99991 | 0.010 | 83.6 ± 2.9 | 0.050 | *66.4 ± 5.8* | 0.250 | 99.6 ± 9.0 | 0.003 | 0.010 |
|  |  | cadusafos | 0.99798 | 0.010 | 106.8 ± 3.0 | 0.100 | 71.4 ± 8.9 | 0.500 | 77.3 ± 6.0 | 0.002 | 0.008 |
|  |  | carbaryl | 0.99971 | 0.010 | 104.7 ± 1.7 | 0.100 | 85.8 ± 2.4 | 0.500 | 104.8 ± 7.2 | 0.003 | 0.010 |
|  |  | carbofuran | 0.99839 | 0.020 | 113.5 ± 2.0 | 0.200 | 97.0 ± 3.1 | 1.000 | 102.9 ± 5.1 | 0.003 | 0.010 |
|  |  | chlorfenvinphos | 0.99894 | 0.010 | 104.6 ± 2.4 | 0.100 | 97.3 ± 4.4 | 0.500 | 105.2 ± 3.8 | 0.003 | 0.010 |
|  |  | chlorpyrifos | 0.99990 | 0.020 | 107.4 ± 3.3 | 0.200 | 91.6 ± 3.7 | 1.000 | 103.8 ± 6.9 | 0.002 | 0.005 |
|  |  | chlorpyrifos-methyl | 0.99956 | 0.005 | 97.5 ± 0.8 | 0.050 | 89.4 ± 5.6 | 0.250 | 98.2 ± 8.0 | 0.003 | 0.010 |
|  |  | coumphos | 0.99811 | 0.010 | 105.3 ± 4.0 | 0.100 | 82.3 ± 4.6 | 0.500 | 96.2 ± 6.3 | 0.006 | 0.020 |
|  |  | cyfluthrin | 0.99594 | 0.010 | 76.5 ± 2.5 | 0.100 | 77.4 ± 3.2 | 0.500 | 94.6 ± 7.2 | 0.003 | 0.010 |
|  |  | cypermethrin | 0.99693 | 0.005 | 78.0 ± 0.1 | 0.050 | *59.1 ± 8.9* | 0.250 | 98.3 ± 6.1 | 0.003 | 0.010 |
|  |  | DEET | 0.99999 | 0.010 | 106.7 ± 2.0 | 0.050 | 90.5 ± 3.5 | 0.250 | 101.6 ± 5.0 | 0.003 | 0.010 |
|  |  | deltamethrin | 0.99893 | 0.010 | 104.1 ± 4.1 | 0.100 | 78.7 ± 1.9 | 0.500 | 95.2 ± 8.3 | 0.002 | 0.005 |
|  |  | diazinon | 0.99997 | 0.010 | 106.6 ± 2.9 | 0.100 | 105.5 ± 3.5 | 0.500 | 107.3 ± 9.1 | 0.002 | 0.005 |
|  |  | dichlorvos | 0.99839 | 0.020 | 113.5 ± 2.0 | 0.200 | 97.0 ± 3.1 | 1.000 | 102.9 ± 5.1 | 0.003 | 0.010 |
|  |  | dicofol | 0.99798 | 0.010 | 106.8 ± 3.0 | 0.100 | 71.4 ± 8.9 | 0.500 | 77.3 ± 6.0 | 0.002 | 0.008 |
|  |  | diflubenzuron | 0.99989 | 0.010 | 106.7 ± 3.3 | 0.100 | 96.7 ± 3.6 | 0.500 | 106.8 ± 7.1 | 0.003 | 0.010 |
|  |  | dieldrin | 0.99998 | 0.001 | 99.8 ± 4.8 | 0.100 | 88.4 ± 11.3 | 0.500 | 108.3 ± 9.7 | 0.003 | 0.010 |
|  |  | dimethoate | 0.99894 | 0.050 | 101.1 ± 1.2 | 0.500 | 95.4 ± 0.98 | 2.500 | 113.0 ± 5.1 | 0.006 | 0.020 |
|  |  | endosulfan sulfate | 0.99990 | 0.010 | 94.7 ± 2.5 | 0.100 | 100.2 ± 5.9 | 0.500 | 103.2 ± 9.0 | 0.002 | 0.005 |
|  |  | endrin | 0.99723 | 0.010 | 94.7 ± 3.3 | 0.100 | 85.6 ± 2.8 | 0.500 | 116.2 ± 2.4 | 0.002 | 0.005 |
|  |  | esfenvalerate | 0.99990 | 0.010 | 85.3 ± 3.9 | 0.100 | 95.6 ± 6.6 | 0.500 | 104.2 ± 6.5 | 0.002 | 0.005 |
|  |  | etoxazole | 0.99834 | 0.010 | 99.3 ± 2.2 | 0.100 | 98.5 ± 6.0 | 0.500 | 198.1 ± 2.4 | 0.002 | 0.008 |
|  |  | ethion | 0.99991 | 0.010 | 104.7 ± 1.7 | 0.100 | 85.8 ± 2.4 | 0.500 | 104.8 ± 7.2 | 0.003 | 0.010 |
|  |  | ethoprophos | 0.99958 | 0.030 | 99.7 ± 3.3 | 0.300 | 95.4 ± 8.2 | 1.500 | 106.3 ± 9.9 | 0.009 | 0.030 |
|  |  | fenazaquin | 0.99997 | 0.010 | 98.8 ± 2.4 | 0.100 | 95.2 ± 5.6 | 0.500 | 109.8 ± 9.2 | 0.002 | 0.005 |
|  |  | fenitrothion | 0.99962 | 0.010 | 94.5 ± 1.1 | 0.100 | 100.9 ± 8.9 | 0.500 | 106.3 ± 4.5 | 0.003 | 0.010 |
|  |  | fenoxycarb | 0.99798 | 0.010 | 106.8 ± 3.0 | 0.100 | 71.4 ± 8.9 | 0.500 | 77.3 ± 6.0 | 0.002 | 0.008 |
|  |  | fenpropathrin | 0.99466 | 0.010 | 101.3 ± 0.6 | 0.100 | 96.8 ± 7.9 | 0.500 | 108.7 ± 8.7 | 0.003 | 0.010 |
|  |  | fenpyroximate | 0.99991 | 0.020 | 114.5 ± 2.4 | 0.200 | 74.5 ± 6.8 | 2.000 | 99.4 ± 8.7 | 0.006 | 0.020 |
|  |  | fenthion | 0.99971 | 0.010 | 104.7 ± 1.7 | 0.100 | 85.8 ± 2.4 | 0.500 | 104.8 ± 7.2 | 0.003 | 0.010 |
|  |  | fenvalerate | 0.99950 | 0.010 | 104.8 ± 3.7 | 0.100 | 82.0 ± 9.5 | 0.500 | 85.4 ± 3.4 | 0.003 | 0.010 |
|  |  | fipronil | 0.99995 | 0.050 | 95.1 ± 3.3 | 0.500 | 79.7 ± 6.4 | 2.500 | 98.5 ± 4.0 | 0.006 | 0.020 |
|  |  | formothion | 0.99986 | 0.010 | 114.5 ± 0.3 | 0.100 | 89.6 ± 11.9 | 0.500 | 106.5 ± 9.1 | 0.002 | 0.005 |
|  |  | gamma-HCH (lindane) | 0.99740 | 0.010 | 83.7 ± 3.5 | 0.100 | 78.7 ± 4.3 | 0.500 | 98.3 ± 7.5 | 0.003 | 0.010 |
|  |  | HCB | 0.99900 | 0.005 | 101.0 ± 3.5 | 0.050 | 93.3 ± 6.0 | 0.250 | 101.3 ± 6.5 | 0.002 | 0.005 |
|  |  | heptachlor | 0.99752 | 0.020 | *125.4 ± 9.4* | 0.200 | 79.7 ± 12.8 | 1.000 | 105.0 ± 7.1 | 0.006 | 0.020 |
|  |  | heptachlor-epoxide | 0.99969 | 0.010 | 106.0 ± 1.4 | 0.100 | 82.6 ± 9.5 | 0.500 | 107.5 ± 6.0 | 0.003 | 0.010 |
|  |  | heptenophos | 0.99834 | 0.010 | 109.3 ± 4.8 | 0.100 | 88.5 ± 5.6 | 0.500 | 106.1 ± 4.4 | 0.003 | 0.010 |
|  |  | hexythiazox | 0.99697 | 0.020 | 104.9 ± 4.6 | 0.200 | 92.3 ± 5.9 | 1.000 | 104.2 ± 8.2 | 0.006 | 0.020 |
|  |  | indoxacarb | 0.99995 | 0.010 | 87.5 ± 2.5 | 0.100 | 92.5 ± 6.8 | 0.500 | 105.2 ± 5.1 | 0.003 | 0.010 |
|  |  | isofenphos | 1.00000 | 0.010 | 104.6 ± 1.6 | 0.100 | 77.7 ± 2.4 | 0.500 | 104.8 ± 5.8 | 0.003 | 0.010 |
|  |  | isofenphos-methyl | 0.99839 | 0.020 | 113.5 ± 2.0 | 0.200 | 97.0 ± 3.1 | 1.000 | 102.9 ± 5.1 | 0.003 | 0.010 |
|  |  | lambda-cyhalothrin | 1.00000 | 0.010 | 70.1 ± 1.4 | 0.100 | 70.8 ± 3.8 | 0.500 | *67.3 ± 4.1* | 0.003 | 0.010 |
|  |  | malathion | 0.99975 | 0.010 | 104.8 ± 1.5 | 0.100 | 89.6 ± 4.9 | 0.500 | 107.4 ± 6.4 | 0.003 | 0.010 |
|  |  | mecarbam | 0.99475 | 0.010 | 72.8 ± 0.8 | 0.100 | 73.7 ± 6.0 | 0.500 | 78. 5 ± 8.3 | 0.003 | 0.010 |
|  |  | methacrifos | 0.99991 | 0.010 | 104.7 ± 1.7 | 0.100 | 85.8 ± 2.4 | 0.500 | 104.8 ± 7.2 | 0.003 | 0.010 |
|  |  | mevinphos | 0.99958 | 0.030 | 99.7 ± 3.3 | 0.300 | 95.4 ± 8.2 | 1.500 | 106.3 ± 9.9 | 0.009 | 0.030 |
|  |  | methidathion | 0.99923 | 0.020 | *137.4 ± 2.1* | 0.200 | 93.0 ± 4.5 | 1.000 | 106.6 ± 6.4 | 0.003 | 0.010 |
|  |  | methoxychlor (DMDT) | 0.99989 | 0.010 | 106.7 ± 3.3 | 0.100 | 96.7 ± 3.6 | 0.500 | 106.8 ± 7.1 | 0.003 | 0.010 |
|  |  | omethoate | 0.99938 | 0.010 | 78.0 ± 2.8 | 0.100 | 73.5 ± 7.7 | 0.500 | *65.5 ± 2.5* | 0.003 | 0.010 |
|  |  | o.p’-DDT | 0.99559 | 0.001 | 73.6 ± 2.2 | 0.100 | 89.5 ± 3.8 | 0.500 | 100.5 ± 8.0 | 0.002 | 0.005 |
|  |  | p.p’-DDD | 0.99697 | 0.002 | 104.9 ± 4.6 | 0.200 | 92.3 ± 5.9 | 1.000 | 104.2 ± 8.2 | 0.003 | 0.020 |
|  |  | p.p’-DDE | 0.99995 | 0.001 | 87.5 ± 2.5 | 0.100 | 92.5 ± 6.8 | 0.500 | 105.2 ± 5.1 | 0.003 | 0.010 |
|  |  | p.p’-DDT | 0.99932 | 0.001 | 108.1 ± 2.5 | 0.100 | *125.1 ± 4.1* | 0.500 | 119.8 ± 8.0 | 0.002 | 0.005 |
|  |  | parathion-ethyl | 0.99463 | 0.050 | 103.1 ± 1.4 | 0.500 | 87.5 ± 9.8 | 2.500 | 103.3 ± 3.7 | 0.003 | 0.010 |
|  |  | parathion-methyl | 0.99771 | 0.010 | 103.4 ± 3.1 | 0.100 | 85.2 ± 4.5 | 0.500 | 103.0 ± 9.1 | 0.003 | 0.010 |
|  |  | permethrin | 0.99922 | 0.030 | 85.3 ± 2.9 | 0.300 | 75.0 ± 5.8 | 1.500 | 105.6 ± 9.5 | 0.009 | 0.030 |
|  |  | phenthoate | 0.99991 | 0.010 | 104.7 ± 1.7 | 0.100 | 85.8 ± 2.4 | 0.500 | 104.8 ± 7.2 | 0.003 | 0.010 |
|  |  | phorate | 0.99424 | 0.010 | 102.5 ± 3.3 | 0.100 | 96.5 ± 2.4 | 0.500 | 107.2 ± 9.2 | 0.003 | 0.010 |
|  |  | phosalone | 0.99906 | 0.010 | 74.0 ± 2.4 | 0.100 | 95.7 ± 3.8 | 0.500 | *59.6 ± 11.29* | 0.003 | 0.010 |
|  |  | phosmet | 0.99975 | 0.010 | 103.0 ± 2.6 | 0.100 | 89.9 ± 2.5 | 0.500 | 103.9 ± 6.2 | 0.003 | 0.010 |
|  |  | phoxim | 0.99968 | 0.005 | 98.7 ± 1.2 | 0.100 | 89.3 ± 8.9 | 0.500 | 99.0 ± 6.0 | 0.003 | 0.010 |
|  |  | pirimicarb | 0.99794 | 0.020 | 109.6 ± 2.7 | 0.200 | 89.2 ± 5.6 | 1.000 | 95.1 ± 13.5 | 0.003 | 0.010 |
|  |  | pirimiphos | 0.99989 | 0.010 | 106.7 ± 3.3 | 0.100 | 96.7 ± 3.6 | 0.500 | 106.8 ± 7.1 | 0.003 | 0.010 |
|  |  | pirimiphos-methyl | 0.99992 | 0.005 | 101.4 ± 0.8 | 0.100 | 94.8 ± 13.6 | 0.500 | 102.4 ± 8.0 | 0.003 | 0.010 |
|  |  | profenofos | 0.99990 | 0.030 | 100.2 ± 0.6 | 0.300 | 87.5 ± 9.7 | 1.500 | 94.2 ± 5.5 | 0.006 | 0.020 |
|  |  | propoxur | 0.99997 | 0.020 | 107.9 ± 2.8 | 0.200 | 87.6 ± 5.2 | 1.000 | 100.4 ± 3.9 | 0.003 | 0.010 |
|  |  | pyridaben | 0.99923 | 0.005 | 107.5 ± 1.8 | 0.100 | 102.0 ± 9.8 | 0.500 | 107.0 ± 6.5 | 0.003 | 0.010 |
|  |  | pyriproxyfen | 0.99994 | 0.010 | 108.0 ± 2.6 | 0.100 | 92.8 ± 6.2 | 0.500 | 108.5 ± 8.5 | 0.003 | 0.010 |
|  |  | quinalphos | 0.99698 | 0.010 | 96.6 ± 0.1 | 0.100 | 93.8 ± 9.0 | 0.500 | 114.2 ± 7.2 | 0.002 | 0.005 |
|  |  | tebufenpyrad | 0.99938 | 0.010 | 78.0 ± 2.8 | 0.100 | 73.5 ± 7.7 | 0.500 | 74.5 ± 6.8 | 0.003 | 0.010 |
|  |  | teflubenzuron | 0.99971 | 0.010 | 104.7 ± 1.7 | 0.100 | 85.8 ± 2.4 | 0.500 | 104.8 ± 7.2 | 0.003 | 0.010 |
|  |  | terbufos | 0.99697 | 0.020 | 104.9 ± 4.6 | 0.200 | 92.3 ± 5.9 | 1.000 | 104.2 ± 8.2 | 0.006 | 0.020 |
|  |  | tetrachlorvinphos | 0.99995 | 0.010 | 87.5 ± 2.5 | 0.100 | 92.5 ± 6.8 | 0.500 | 105.2 ± 5.1 | 0.003 | 0.010 |
|  |  | tetradifon | 0.99991 | 0.020 | 114.5 ± 2.4 | 0.200 | 74.5 ± 6.8 | 2.000 | 99.4 ± 8.7 | 0.006 | 0.020 |
|  |  | thiacloprid | 0.99991 | 0.010 | 104.7 ± 1.7 | 0.100 | 85.8 ± 2.4 | 0.500 | 104.8 ± 7.2 | 0.003 | 0.010 |
|  |  | thiamethoxam | 0.99958 | 0.030 | 99.7 ± 3.3 | 0.300 | 95.4 ± 8.2 | 1.500 | 106.3 ± 9.9 | 0.009 | 0.030 |
|  |  | triazophos | 0.99968 | 0.005 | 98.7 ± 1.2 | 0.100 | 89.3 ± 8.9 | 0.500 | 99.0 ± 6.0 | 0.002 | 0.005 |
|  |  | zeta-cypermethrin | 1.00000 | 0.010 | 103.8 ± 1.1 | 0.100 | 71.0 ± 9.9 | 0.500 | 87.0 ± 7.2 | 0.003 | 0.010 |

R^2^ – correlation coefficient;

italics – pesticides with recovery <70% and >120%.

fort. – fortification
