# Supplementary material for: Studies of pesticide residues in tomatoes and cucumbers from Kazakhstan and the associated health risks
Source: Environ Monit Assess. 2015 Sep 4;187(10):609. doi: 10.1007/s10661-015-4818-6 (PMC4559566; doi:10.1007/s10661-015-4818-6)
Supplement: Supplementary file 2 — (RTF 271 kb) [file 10661_2015_4818_MOESM2_ESM.rtf]

Table S2 Results of participation in proficiency testing (PT).

	Pesticide	Assigned value 
(mg kg-1)	Laboratory results (mg kg-1)	z-score	

Potato homogenate 
EUPT-FV15
European Commission - University of Almeria (Spain)
2013
	
1	azoxystrobin	0.203	0.204	0.0	
2	chlorothalonil	0.160	0.155	-0.1	
3	chlorpropham	1.700	1.292	-1.0	
4	cypermethrin	0.100	0.105	0.2	
5	diazinon	0.195	0.204	0.2	
6	iprovalicarb	0.090	0.105	0.7	
7	pencycuron	0.269	0.265	-0.1	
8	prochloraz	0.058	0.061	0.2	
9	procymidone	0.110	0.126	0.6	
10	thiabendazole	1.710	1.462	-0.6	

Potato homogenate 
EUPT-SRM8
European Commission - University of Almeria (Spain)
2013
	
1	captan	1.010	0.920	-0.36	
2	dicofol	1.030	0.860	-0.66	
3	folpet	1.320	1.350	0.09	

Pear homogenate 
EUPT-FV14
European Commission - University of Almeria (Spain)
2012
	
1	boscalid	0.177	0.183	0.1	
2	cyprodinil	0.247	0.251	0.1	
3	diazinon	0.050	0.064	0.8	
4	diphenylamine	0.188	0.193	0.1	
5	fludioxonil	0.171	0.171	0.0	
6	folpet	0.413	0.423	0.1	
7	indoxacarb	0.083	0.085	0.1	
8	iprodione	0.334	0.341	0.1	
9	phosmet	0.068	0.076	0.5	
10	pyraclostrobin	0.172	0.163	-0.2	
11	pyrimethanil	0.107	0.111	0.1	
12	thiabendazole	0.461	0.519	0.5	
13	thiacloprid	0.030	0.030	0.0	

Apple Purée
FAPAS Proficiency Test 19115
FAPAS Central Science Laboratory, Sand Hutton, York UK -
UKAS Proficiency Testing Provider No 0009
2011
	
1	acrinathrin	0.288	0.245	-0.8	
2	p,p'-DDE	0.027	0.0238	-0.5	
3	paclobutrazol	0.260	0.0268	0.1	
4	triadimefon	0.174	0.169	-0.1	
5	vinclozolin	0.144	0.150	0.2	

Mandarin homogenate 
EURL-PT-FV-13
European Commission - University of Almeria (Spain)
2011
	
1	chlorpyrifos	0.786	0.833	0.2	
2	deltamethrin	0.133	0.125	-0.2	
3	diazinon	0.189	0.188	0.0	
4	imazalil	1.30	1.280	-0.1	
5	indoxacarb	0.792	0.725	-0.3	
6	malathion	0.381	0.436	0.6	
7	methidathion	0.730	0.862	0.7	
8	pendimethalin	0.583	0.607	0.2	
9	phosalone	0.280	0.266	-0.2	
10	prochloraz	0.301	0.248	-0.7	
11	pyriproxyfen	0.443	0.420	-0.2	
12	tolylfluanid	0.900	0.922	0.1	

Cucumber  Purée
FAPAS Proficiency Test 19104 
FAPAS Central Science Laboratory, Sand Hutton, York UK -
UKAS Proficiency Testing Provider No 0009
2010
	
1	chlorpyrifos	0.0837	0.0851	-0.3	
2	fenvalerate  	0.1730	0.1973	0.3	
3	pirimiphos-methyl	0.0809	0.0855	0.7	
4	triazophos	0.3540	0.3333	0.1	

Leek homogenate 
EURL-PT-FV-12
European Commission - University of Almeria (Spain)
2010
	
1	azinphos-methyl	0.048	0.051	0.3	
2	chlorothalonil	0.216	0.326	1.9	
3	chlorpyrifos	0.176	0.200	0.5	
4	ethion	0.071	0.067	-0.2	
5	fenpropathrin	0.062	0.066	0.3	
6	kresoxim-methyl	0.316	0.338	0.3	

Apple Purée
FAPAS Proficiency Test 1987
FAPAS Central Science Laboratory, Sand Hutton, York UK -
UKAS Proficiency Testing Provider No 0009
2009
	
1	boscalid	0.256	0.2599	0.1	
2	buprofezin	0.169	0.1888	0.6	
3	ethion	0.204	0.2437	1.0	
4	metalaxyl	0.184	0.195	0.3	
5	parathion-ethyl	0.249	0.2836	0.7	
6	triadimefon	0.322	0.3222	0.0	

Cauliflower homogenate 
EURL-PT-FV-11
European Commission - University of Almeria (Spain)
2009
	
1	azinphos-methyl	0.355	0.5057	1.7	
2	boscalid	0.414	0.384	-0.3	
3	buprofezin	0.638	0.7406	0.6	
4	carbofuran	0.283	0.23	-0.7	
5	deltamethrin	0.157	0.2189	1.6	
6	diazinon	1.25	1.64	1.2	
7	isofenphos-methyl	0.540	0.5466	0.0	
8	lambda cyhalothrin	0.266	0.3904	1.9	
9	metalaxyl	0.450	0.44	-0.1	
10	methidathion	0.472	0.5636	0.8	
11	phosalone	0.368	0.5046	1.5	
12	procymidone	0.780	0.815	0.2	
13	triazophos	0.538	0.5356	0.0	

Pear  Purée
FAPAS Proficiency Test 1991 
FAPAS Central Science Laboratory, Sand Hutton, York UK -
UKAS Proficiency Testing Provider No 0009
2009
	
1	boscalid	0.344	0.4461	1.6	
2	bupirimate	0.149	0.1691	0.6	
3	iprodione	0.539	0.6367	1.0	
4	permethrin	0.0475	0.0481	0.1	
5	trifloxystrobin	0.278	0.3209	0.8	

Apple Purée
FAPAS Proficiency Test 1975 
FAPAS Central Science Laboratory, Sand Hutton, York UK -
UKAS Proficiency Testing Provider No 0009
2008
	
1	bupirimate	0.136	0.1683	1.1	
2	chlorpyrifos	0.0457	0.0643	1.8	
3	pirimicarb	0.0702	0.0766	0.4	

Tomato Purée
FAPAS Proficiency Test 1980
FAPAS Central Science Laboratory, Sand Hutton, York UK -
UKAS Proficiency Testing Provider No 0009
2008
	
1	parathion-methyl	0.1569	0.1758	0.6	
2	penconazole	0.0754	0.0778	0.1	
3	trifloxystrobin	0.2221	0.2369	0.3	
4	vinclozolin	0.1758	0.1915	0.4	
